# Targeting cell death mechanisms: the potential of autophagy and ferroptosis in hepatocellular carcinoma therapy

**DOI:** 10.3389/fimmu.2024.1450487

**Published:** 2024-09-09

**Authors:** Beibei Liu, Ling Liu, Yang Liu

**Affiliations:** ^1^ Department of Radiology and Huaxi MR Research Center (HMRRC), Functional and Molecular Imaging Key Laboratory of Sichuan Province, West China Hospital, Sichuan University, Chengdu, Sichuan, China; ^2^ Division of Biliary Surgery, Department of General Surgery, West China Hospital, Sichuan University, Chengdu, Sichuan, China; ^3^ Day Surgery Center, General Practice Medical Center, West China Hospital, Sichuan University, Chengdu, Sichuan, China

**Keywords:** autophagy, ferroptosis, cancer progression, drug resistance, cell death, tumor

## Abstract

Ferroptosis is a type of cell death that plays a remarkable role in the growth and advancement of malignancies including hepatocellular carcinoma (HCC). Non-coding RNAs (ncRNAs) have a considerable impact on HCC by functioning as either oncogenes or suppressors. Recent research has demonstrated that non-coding RNAs (ncRNAs) have the ability to control ferroptosis in HCC cells, hence impacting the advancement of tumors and the resistance of these cells to drugs. Autophagy is a mechanism that is conserved throughout evolution and plays a role in maintaining balance in the body under normal settings. Nevertheless, the occurrence of dysregulation of autophagy is evident in the progression of various human disorders, specifically cancer. Autophagy plays dual roles in cancer, potentially influencing both cell survival and cell death. HCC is a prevalent kind of liver cancer, and genetic mutations and changes in molecular pathways might worsen its advancement. The role of autophagy in HCC is a subject of debate, as it has the capacity to both repress and promote tumor growth. Autophagy activation can impact apoptosis, control proliferation and glucose metabolism, and facilitate tumor spread through EMT. Inhibiting autophagy can hinder the growth and spread of HCC and enhance the ability of tumor cells to respond to treatment. Autophagy in HCC is regulated by several signaling pathways, such as STAT3, Wnt, miRNAs, lncRNAs, and circRNAs. Utilizing anticancer drugs to target autophagy may have advantageous implications for the efficacy of cancer treatment.

## Highlights

- The paper highlights the critical involvement of ferroptosis, a form of regulated cell death driven by iron-dependent lipid peroxidation, and autophagy, a cellular degradation and recycling process, in the progression and therapeutic response of HCC.
- It outlines the significant impact of non-coding RNAs in modulating both ferroptosis and autophagy within HCC cells, acting either as oncogenes or suppressors, thus affecting tumor growth, metastasis, and drug resistance.
- The document elaborates on the molecular pathways and mechanisms, such as the PI3K/AKT/mTOR signaling pathway, through which autophagy and ferroptosis influence HCC development, including their roles in proliferation, metastasis, energy metabolism, and chemotherapy resistance.
- The paper discusses the therapeutic potential of targeting autophagy and ferroptosis in HCC, highlighting specific compounds and strategies that induce or inhibit these processes to suppress tumor growth and overcome drug resistance, including the use of traditional Chinese Medicine and novel targeted drugs.
- It calls for further research into the detailed signal transduction pathways and key transcriptional regulators of ferroptosis and autophagy in HCC, aiming to develop targeted therapeutic strategies to enhance treatment efficacy and overcome resistance.

## Questions

- How do non-coding RNAs regulate ferroptosis and autophagy in hepatocellular carcinoma?
- What are the dual roles of autophagy in hepatocellular carcinoma, and how do they affect tumor growth and treatment response?
- Which molecular pathways are involved in regulating autophagy and ferroptosis in HCC, and how do they influence cancer progression and drug resistance?
- What therapeutic strategies are being explored to target autophagy and ferroptosis in HCC, and what potential do they hold for improving treatment outcomes?
- What future research directions are suggested for understanding the complex roles of ferroptosis and autophagy in HCC, and how could this knowledge contribute to the development of novel therapeutic targets and strategies?

## 1 Introduction

On a global scale, primary liver cancer ranks second in terms of mortality and seventh in terms of cancer incidence (1–3). The continents of Asia and Africa illustrate the most elevated prevalence level of liver cancer (4). Mongolia has the greatest incidence rate of primary liver cancer, with 93.7 cases per 100,000 people. China has the biggest number of patients due to its noticeable incidence level of 18.3 cases per 100,000 people and its population of 1.4 billion individuals. Hepatocellular carcinoma (HCC) is the most common type of liver cancer, representing up to 75% of all cases (4). The incidence rate of HCC has been decreasing in many regions with formerly high rates, while the opposite trend has been documented in areas with low incidence rates (5). From 1978 to 2012, the occurrence rate of HCC declined in certain regions including Italy and Asian countries, while it rose in the United States, India, Oceania, and several European countries (5). Nevertheless, there has been a decline in the number of HCC cases in the United States in recent years (6, 7). Age, gender, and race are determinants that impact the probability of getting HCC. HCC represents a direct correlation with age, namely up to 75 years (4). Men have an incidence rate of HCC that is two to four times higher than women (4). HCC is ranked as the sixth most prevalent malignancy globally, with over 500,000 new cases detected each year. This high incidence contributes to a significant mortality rate, making HCC the third leading cause of death among all types of cancers (8). The estimated incidence rate of HCC is 3.6-10.5 cases per 100,000 people, with a potential increase to 16 cases per 100,000 worldwide (9, 10). The prognosis for patients with HCC is generally poor, with about 5% of individuals surviving beyond 5 years following diagnosis. This pertains to the delayed detection of HCC patients, with only a mere 15% of individuals meeting the criteria for liver transplantation and surgical intervention. Half of them undergo non-surgical therapy, whereas 35% or more obtain the optimal treatment during diagnosis (10). HCC can be caused by a range of risk factors, such as alcohol intake, hepatitis virus infection, cirrhosis, and nonalcoholic fatty liver disease (11).

Molecular-level interaction among a number of pathways and mechanisms governs the progression of HCC. Met phosphorylates Fis1, which facilitates phosphorylation of Fis1 and stimulates HCC migration and fission (12). YAP expression can be increased by CD44, which speeds up the progression of HCC (13). The progression of HCC appears to be significantly influenced by adiposity. Insulinemia, steatosis, and the concentration of inflammatory cytokines are all diminished as PI3Kγ ablation inhibits HCC proliferation (14). In order to facilitate tumor metastasis, ZRANB1, in its capacity as a deubiquitinate, promotes LOXL2 expression, thereby contributing to the progression and malignancy of HCC (15). DEAH-box polypeptide 32 stimulates β-catenin signaling in order to promote tumor growth (16). Stimulation of Wnt/β-catenin signaling has been shown to be associated with HCC progression (11). HCC progression is facilitated by CircZFR, which increases HMGA2 expression via miR-375 sponging (17). The prognostic value of NTF3, which is limited in expression in HCC, is debatable. In addition, NFT3 promotes the overabundance of immune cells within HCC, such as natural killer cells, CD4+ cells, mast cells, and macrophages (18). ADORA2A-AS1, on the other hand, inhibits the FSCN1/Akt axis in order to promote apoptosis and impede tumor progression *in vivo* and *in vitro* (19). This factor may influence the progression of HCC.

In the recent years, the function of non-coding RNAs in the regulation of HCC progression has been of importance. miR-612 can enhance RSL3-mediated ferroptosis through regulation of mevalonate pathway to impair progression of HCC (20). A combination of curcumin and berberine can increase SOX11 levels through miR-221 downregulation for reducing HCC growth (21). In addition, lncRNA NEAT1 impairs senescence in HCC through KIF11-related CDKN2A downregulation (22). LncRNA FTO-IT1 has been shown to enhance glycolysis and malignancy of HCC through enhancing levels of GLUT1 and PKM2 (23). LncRNA GBAP1 can be upregulated by METTL3 to overexpress BMP/SMAD in enhancing progression of HCC (24). CircRNA-0004018 is a prognostic and reliable biomarker in HCC (25) and circRNAs can regulate therapy resistance and progression of HCC cells (26, 27). Noteworthy, the non-coding RNAs have been shown to regulate autophagy and ferroptosis in HCC (28–30). Silencing circRNA 0016142 can stimulate ferroptosis in HCC cells through reducing GPX4 levels (31). Loading miR-654-5p into extracellular vesicles can stimulate ferroptosis through HSPB1 downregulation to mediate sorafenib sensitivity in HCC (32). The study develops a dynamic Boolean network model to explore interactions between PTEN, PTENP1, and miR-21 in cancers like breast cancer, HCC, and OSCC. The model aligns with experimental observations, detailing how DNA damage response (DDR) triggers cellular processes such as cell cycle arrest, senescence, autophagy, apoptosis, drug resistance, and EMT. It highlights PTENP1’s role in inhibiting miR-21 and enhancing PTEN’s function, which is critical for autophagy and DDR outcomes. The research also identifies feedback loops that provide new insights into potential therapeutic targets for promoting autophagy and overcoming drug resistance in cancer (33). In the present review, our aim is to highlight the role of autophagy and ferroptosis in the regulation of HCC progression in terms of controlling the hallmarks of HCC and determining the response of tumor cells to therapy. Furthermore, autophagy and ferroptosis demonstrate interaction and therefore, such interaction is described in HCC to further direct the future studies in the development of novel therapeutics for this malignant disease.

## 2 Autophagy machinery

**Figure 1:**
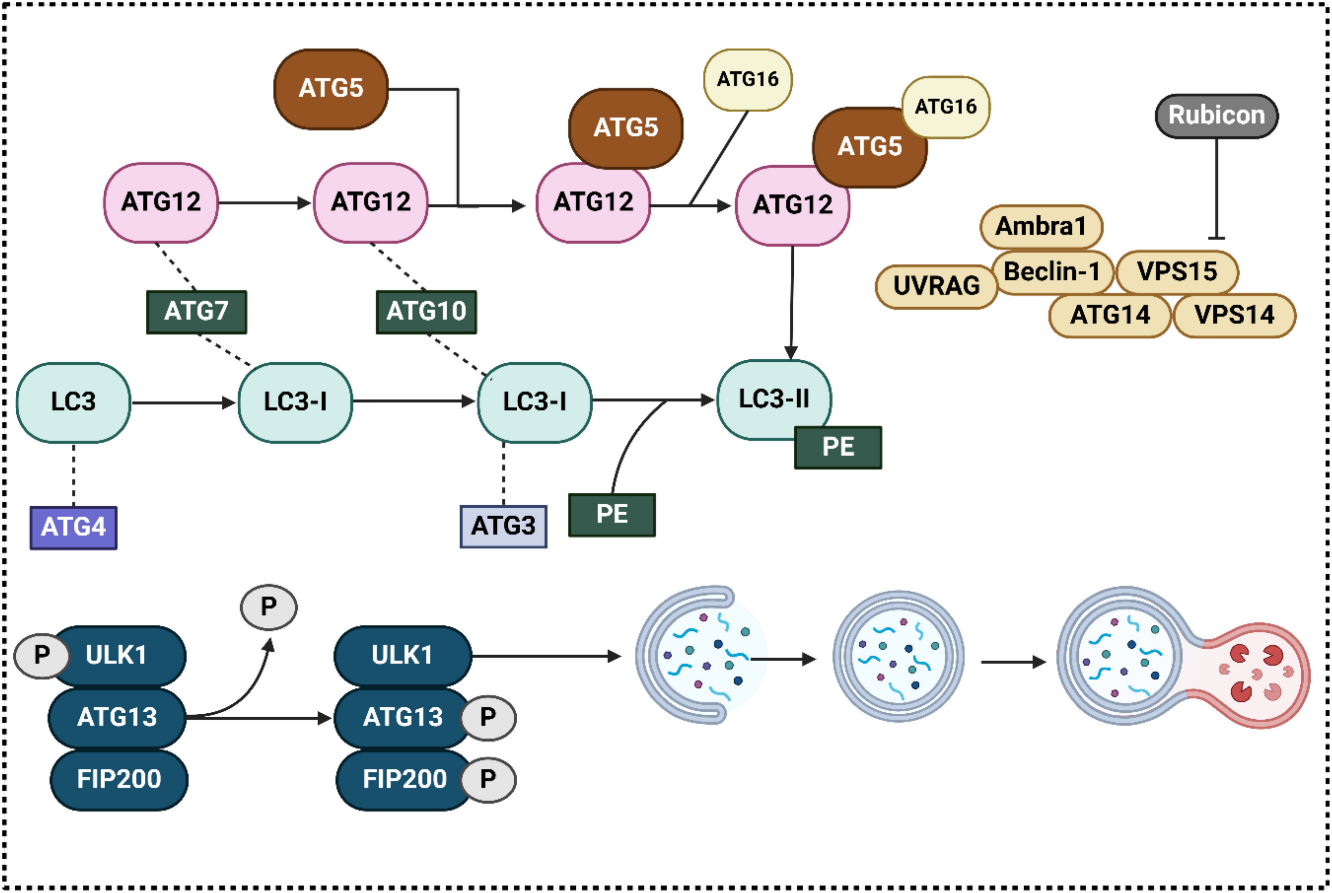
The regulators of autophagy in the cells.

Initiation, elongation, creation of autophagosomes, autophagosome fusion with lysosomes, and finally destruction are the successive processes that make up autophagy, which is a highly conserved process (34–36). In autophagy, macroautophagy, microautophagy, and chaperone-mediated autophagy are the three kinds (37, 38). Autophagy participates in the degradation of long-lived proteins and organelles (39). These stages are controlled by genes that are connected to autophagy, also known as Atgs. To this day, more than thirty Atgs have been identified, and careful consideration has been given to the roles that they perform. In particular, research conducted in the liver utilizing specific Atg-deletion models give evidence of the crucial functions that autophagy functions in the body’s adaptive reactions to stress and famine, additionally in the processes of cellular differentiation, development, and homeostasis (40–46). In what follows, we will go over the autophagy process in detail. At the outset, the initiation step is regulated by the AMPK, ULK1, and mTORC1 complexes. The most fundamental factor in hindering the development of autophagosomes by ULK1 is mTORC1. In the presence of glucose, which is a starved food, active AMPK blocks mTORC1, which in turn directly phosphorylates ULK1, and initiates autophagy. (47, 48); (ii) Beclin-1, Vps34, p150, Atg14L/Barkor, and Ambra-1 form a complex that mediates phagophore nucleation and is a class III phosphatidylinositol 3-kinase (PI3K). (49); (iii) Two complexes attached to ubiquitin-like proteins regulate the phagophore’s elongation into a full autophagosome: Alphabet 5–Atg12–Atg16L1 and LC3–II (50–52). These processes need the presence of a number of Atgs, such as Atg7E1-like protein, Atg 10, andE2-like protein, which are some of the essential mediators. The most important mammalian homologue of Atg8 is called LC3. After the fusion of autophagosomes and lysosomes, LC3-1 undergoes a transformation into LC3-II and is then destroyed (53). It is for this reason that LC3-II is regarded as an autophagosome marker (**Figure 1**) (54). During the process of microautophagy, cargo can be ingested either randomly or by targeting each cargo molecule individually, depending on the level of selection required (55). The last stage of microautophagy is autophagic breakdown. In light of the fact that there are numerous evaluations that are of high quality about the autophagy process, reading those reviews will be beneficial for gaining a deeper comprehension of the autophagic pathway (56, 57). In the recent years, the function of autophagy in different human cancers has been evaluated including breast cancer (58), pancreatic cancer (59) and prostate tumor (60), among others. Furthermore, autophagy has been determined to regulate the response of tumor cells to chemotherapy (61, 62).

Autophagy primarily functions to maintain cellular homeostasis by removing damaged organelles and proteins, thereby preventing the accumulation of cellular debris and supporting metabolic stability. This aspect of autophagy plays a critical role in tumor suppression, particularly in the early stages of cancer development. By eliminating damaged mitochondria and reducing oxidative stress, autophagy prevents the accumulation of mutations and chromosomal instabilities that can lead to tumorigenesis. Furthermore, autophagy can mediate the degradation of oncoproteins and modulate inflammation within the tumor microenvironment, thereby exerting a protective effect against the initiation and progression of cancer. However, the effectiveness and extent of these tumor-suppressive functions can vary significantly based on genetic and environmental contexts, which influence the regulatory pathways and effectiveness of autophagic processes. Conversely, in established tumors, especially in the harsh environments characterized by hypoxia and nutrient depletion commonly found in solid tumors like HCC, autophagy can promote tumor growth and survival. In such scenarios, autophagy provides an alternative source of metabolic substrates through the recycling of intracellular components, supporting cellular metabolism and enabling cancer cells to survive under metabolic stress. This adaptive survival mechanism allows tumor cells to cope with the increased metabolic demands of rapid growth and proliferation. Moreover, autophagy has been implicated in resistance to chemotherapy, as the process can remove damaged organelles and proteins induced by treatment, thereby reducing drug-induced apoptosis and enhancing the resilience of cancer cells. The transition of autophagy from a tumor-suppressive to a tumor-promoting role is influenced by several factors including the stage of cancer, mutation status of key oncogenes and tumor suppressors (such as p53, PTEN, and PI3K), and the specific metabolic and microenvironmental conditions of the tumor. For instance, in cells with defective apoptosis mechanisms, autophagy often serves as an alternative cell death pathway; however, in cells where key autophagy genes are mutated or deleted, such as BECN1 or ATG5, autophagy may fail to execute its tumor-suppressive role effectively. Moreover, the interaction between autophagy and other cell death pathways, such as necroptosis and ferroptosis, further complicates its role in cancer. These interactions can influence the overall outcome of cancer therapy, with implications for the development of resistance to treatments that induce cell death by targeting these pathways. Additionally, the crosstalk between autophagy and immune responses within the tumor microenvironment plays a crucial role in modulating the immune surveillance of tumors and the efficacy of immunotherapies. Given these complexities, the effectiveness of therapies targeting autophagy in HCC can vary significantly among patients. It is crucial to consider the specific genetic alterations, stage of disease, and microenvironmental factors when designing and applying autophagy-modulating therapies. Personalized approaches that take into account the heterogeneity of tumor biology and the dual roles of autophagy could enhance the efficacy of these treatments. Future research should focus on delineating the conditions under which autophagy acts as a tumor suppressor versus a promoter, utilizing advanced genetic, proteomic, and metabolomic analyses to better understand the regulatory networks at play. This knowledge is essential for the strategic design of intervention points that can either inhibit or induce autophagy to achieve therapeutic benefits in HCC. While the dual roles of autophagy in cancer offer promising targets for therapeutic intervention, an overly simplified view fails to capture the full therapeutic potential and risks of targeting this complex cellular process. A deeper understanding of the molecular and contextual factors that dictate autophagy’s role across different stages of cancer and patient backgrounds is paramount for the successful integration of autophagy-modulating strategies in the clinical management of HCC.

## 3 Autophagy in hepatocellular carcinoma progression

Proliferation, metastasis, energy metabolism, resistance to chemotherapy and radiation, and autophagy are just a few of the many biological processes influenced by the PI3K/AKT/mTOR, a classic dysregulated pathway in hepatocarcinogenesis (63–65). New insights into the progression of HCC mediated by TAMs and the identification of new therapeutic targets could be achieved by learning about the involvement of miR-210 and the PI3K/AKT/mTOR. In this respect, Bi and co-workers exhibited that via PI3K/AKT/mTOR, miR-210 enhances hepatocellular carcinoma progression by regulating macrophage autophagy (66). The study revealed that miR-210 expanded expression in M2 macrophages, extending autophagy-related gene and protein expression. However, apoptosis-related proteins reduced. MDC staining and transmission electron microscopy illustrated the accumulation of autophagosomes in the miR-210 mimic group. HCC cells co-cultured with miR-210 mimics represented elevated proliferation and invasive ability, while apoptosis rates were reduced. Promoting or impeding autophagy could raise or abolish these impacts. In this connection, Toshima and colleagues revealed that the activation of mitochondrial β-oxidation by autophagy accelerates the progression of hepatocellular cancer (67). The results showed that LC3 expression is highly elevated in HCC tissues, correlated with HIF1α expression and tumor size. This expression predicts HCC recurrence after surgery, especially in large tumors. Huh7 treated with autophagy-inhibitor under hypoxia has lower viability because mitochondrial β-oxidation is hindered. Promoting HIF1α-mediated proliferation, autophagy is observed in HCC. by maintaining intracellular ATP, depending on mitochondrial β-oxidation activation.

In their investigation of autophagy’s function in cancer development (68), Takamura and colleagues utilized animals lacking the ATG5 gene in their livers (44). They discovered that inhibiting ATG5 expression resulted in oxidative DNA damage, a decrease in liver autophagy and the development of benign tumors in the liver that do not appear to be cancerous. The failure to form HCC was associated with the activation of tumor suppressors, including TP27, TP21, TP16, andTP53. These suppressors hindered the development of tumors by impeding autophagy, which manifested as TP62 accumulation, mitochondrial swelling, oxidative stress, and responses to genomic damage (69). Concurrently, Mice lacking ATG7 in the liver grew tumors that were less in size. following TP62 deletion (44, 69), suggesting that autophagy deficiency-induced p62 accumulation contributes to tumor progression. The m6A mutation has been linked to multiple biological processes, including stem cell differentiation, tissue formation, and tumor progression, according to mounting evidence. In this regard, Li and colleagues exhibited that hypoxia-induced autophagy and the aggressiveness of hepatocellular carcinoma are caused by the HIF-1α-induced expression of the m6A reader YTHDF1, which promotes the translation of ATG2A and ATG14 (70). The results demonstrated that YTHDF1 aided autophagy and autophagy-related HCC via binding to m6A-modified mRNA of ATG2A and ATG14, two genes involved in autophagy. All things considered, the expression of YTHDF1 generated by HIF-1α was linked to hypoxia-induced autophagy and the advancement of HCC connected to autophagy through the promotion of translation of ATG2A and ATG14, two genes involved in autophagy, in a way that was dependent on m6A.

It has been found that circRNAs are involved in several tumor-malignant activities in HCC. Therefore, Wang and colleagues revealed that hepatocellular carcinoma progression is hastened by exosomal circTGFBR2 via an upregulation of ATG5-mediated protective autophagy (71). Based on the consequences, ircTGFBR2, which is transported into HCC cells through exosomes, acts as a competing endogenous RNA. It binds to miR-205-5p, which in turn enhances ATG5 production and autophagy in HCC cells, making them resistant to starvation. Through the circTGFBR2/miR-205-5p/ATG5 axis, researchers indicated that circTGFBR2 is an innovative tumor promoter circRNA in hepatocytic exosomes that accelerates HCC growth by amplifying ATG5-mediated protective autophagy. Autophagy is necessary for the transformation of benign hepatic lesions into malignant HCC, according to a more recent study by Liu and colleagues (72). The results of their study indicated that the inhibition of TP53 and the stimulation of NANOG transcription factor expression were crucial for the maintenance of hepatic cancer stem cells and the facilitation of hepatocarcinogenesis, mitophagy, and autophagy (72). TAutophagy is crucial for cancer cell survival when tumors, such as HCC, have grown., whereas it initially functions as a tumor suppressor in nontumor cells or during the early phases of tumor cell development. Autophagy is necessary to promote tumorigenesis in cells expressing oncogenic Ras (73, 74), and maintains oxidative metabolism or facilitates glycolysis, both of which are mechanisms within solid tumors, autophagy has been demonstrated to improve tumor cell survival in hypoxic areas (75).

**Figure 2:**
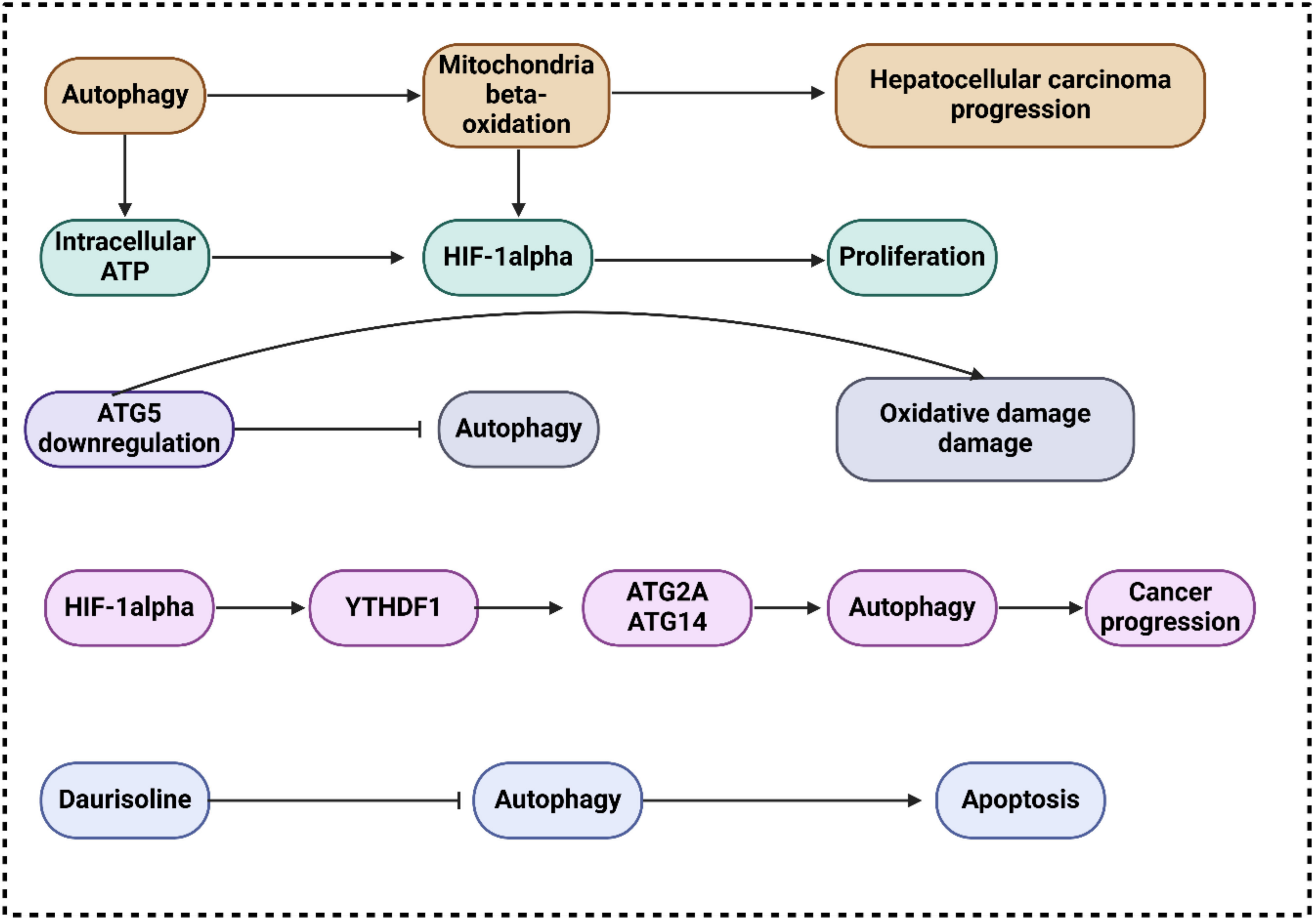
The function of autophagy in hepatocellular carcinoma progression.

In another research, Gao and colleagues exhibited that the progression of IL-1β-mediated hepatocellular carcinoma is accelerated through inflammasome accumulation and self-recruitment when autophagy is inhibited in macrophages (76). By increasing IL-1β release through NLRP3 inflammasome accumulation and by macrophage self-recruitment through the CCL20 signaling pathway, the findings demonstrated that inhibiting tumor macrophage autophagy enhanced the advancement of HCC. One potentially viable therapeutic option for patients with HCC is the interruption of this loop that promotes metastasis by the inhibition of IL-1β. In their research, Xue and collaborators exhibited that Daurisoline promotes cispaltin-induced cell death and slows the growth of hepatocellular cancer by limiting autophagy (77). Whenever DAS was applied to HCC cells, a marked reduction in the mature forms of cathepsin B and cathepsin D was noted. In addition, the anti-cancer effects of cisplatin (cDDP) on HCC cells were significantly increased by DAS therapy, as shown by the considerable reduction of cell viability and proliferation and the enhancement of apoptosis. In addition, the nude mouse xenograft models with HCC demonstrated that the combination of DAS and cDDP considerably slowed tumor progression *in vivo* when compared to the cDDP alone group (**Figure 2**).

## 4 Autophagy in the regulation of hepatocellular carcinoma chemoresistance

Treatments for advanced HCC patients involve induction or inhibition of autophagy; however, drug resistance continues to pose a significant obstacle (78). Chemotherapeutic medications have the potential to augment autophagic flux, thereby potentially bolstering drug resistance and cell survival (79). Autophagy is a mechanism facilitating the survival of cancer cells. Treatment resistance is expanded by autophagy induction, whereas cell mortality is extended by autophagy prevention (80–82). Cancer drug resistance is influenced by various signaling pathways and crucial regulators. Autophagy, in particular, is under the control of proto-oncogenes, tumor suppressors, and noncoding RNAs (83, 84). Autophagy inhibitors can circumvent therapeutic resistance induced by autophagy inducers (81). miR-520b suppressed ATG7-dependent autophagy and enhanced doxorubicin sensitivity in HCC. according to a study by Gao and colleagues (85). Based on these findings, the miR-520b/ATG7 pathway may represent a promising avenue for chemosensitive treatment of HCC. In this context, Zhao and colleagues represented that through the P38/Hsp27/CREB/ATG7 pathway, CD13 [romotes chemoresistance in hepatocellular carcinoma cells by inducing autophagy (86). T An increase in CD13 levels activates the P38/HSP27/cAMP response element-binding protein (CREB) signaling pathway, which in turn decreases the effectiveness of cytotoxic drugs. To make HCC cells more sensitive to 5-fluorouracil, P38 or CREB blockage is used. By establishing a connection with the ATG7 promoter, CREB enhances autophagy and chemoresistance in HCC cells. Inhibiting CD13 expression reduces autophagy, tumor cell proliferation, and ATG7 expression in living organisms. PADI4 is a type of PAD enzyme found in different cells, such as breast cancer cells, leukocytes, and embryonic stem cells and is involved in cancer etiology and rheumatoid arthritis (87, 88). It is expressed in adenocarcinoma and non-adenocarcinoma tumors, suggesting its role in tumorigenesis (89). It has not been acknowledged, however, that PADI4 is involved in HCC cell chemoresistance. Thus, Fan and collaborators represented that through activating autophagy, peptidylarginine deiminase IV contributes to the development of chemoresistance in hepatocellular cancer (90). The consequences demonstrated that chemoresistance is linked to increased PADI4 expression in HCC patients who experienced TACE following surgery. Furthermore, researchers discovered that chemotherapeutic drug resistance occurred in both *in vitro* and *in vivo* when PADI4 was overexpressed in HCC cell lines. Curiously, it was found that HCC cells overexpressing PADI4 went through autophagy, a process that cells use to fight against the cell death caused by chemotherapy. Both *in vitro* and *in vivo* studies have shown that autophagy inhibitors can restore chemotherapeutic sensitivity to HCC cells.

**Figure 3:**
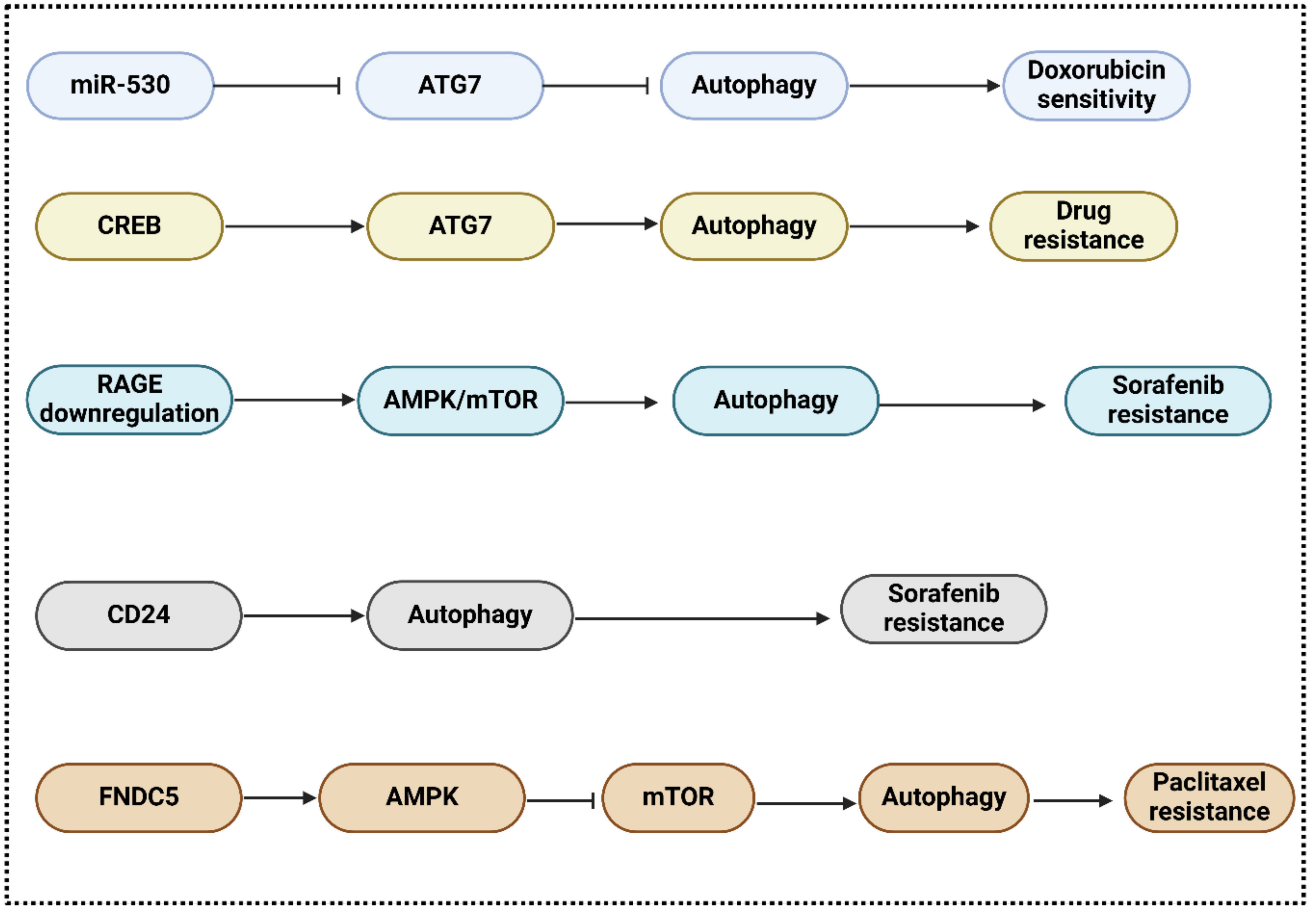
The function of autophagy in the regulation drug resistance.

Sorafenib, which induces autophagy and has a substantial extended overall survival rate in HCC patients, is the only systemic agent presently approved for the treatment of advanced-stage HCC (91). With approximately 30% of patients undergoing HCC stages III/IV responding to sorafenib, the emergence of intrinsic and acquired resistance to the drug continues to pose a significant prognostic challenge. Autophagic induction with prosurvival properties by sorafenib may constitute the potential fundamental mechanism (92). In contrast, the efficacy of sorafenib in combination with everolimus, a highly potent inhibitor of mTOR, was not supported by data from a recent multinational phase II trial that was randomized and performed across multiple centers (93). RAGE modulates autophagy and is responsible for promoting HCC proliferation and sorafenib resistance, according to a recent study. RAGE deficiency activates the AMPK/mTOR signaling pathway, which further contributes to the sorafenib response. These findings suggest that Rage could serve as a biomarker of sorafenib resistance and a potential therapeutic target in HCC (94). Metastasis, angiogenesis, proliferation, apoptosis, and apoptosis of HCC cells are all noticeably affected by RAGE ligands, such as considerable mobility group box 1 (HMGB1) (95, 96). In multiple tumor types, including neuroblastoma, osteosarcoma, and lung cancer, HMGB1 additionally is involved in chemotherapy resistance. Furthermore, a recent study proposed that HMGB1 may play an unexpected role in regulating resistance to sorafenib therapy in HCC; this suggests that HMGB1 and HCC share a positive association (97). In addition, sorafenib resistance in HCC is regulated by the cell surface molecule CD24 through the activation of autophagy; hence, sorafenib sensitivity increased significantly when CD24 was depleted or autophagy was inhibited (98). Furthermore, Zhou and colleagues in their investigation approved that in hepatocellular carcinoma cells, fibronectin type III domain-containing protein 5 stimulates autophagy through the AMPK/mTOR signaling pathway, which adds to the chemoresistance to nab-paclitaxel (99). The results showed that hepatocellular carcinoma tissues have higher FNDC5 expression than normal tissues, which can be reduced by knockdown or overexpression of FNDC5. Overexpression increases resistance to treatment and promotes autophagy via the AMPK/mTOR signaling pathway, reducing cell death induced by nab-paclitaxel. In addition, insulin resistance (IR) and type 2 diabetes mellitus (T2DM) are two independent risk factors for the significant mortality level of HCC patients. IR and T2DM can be instigated by a variety of pathological events, such as inflammation and carcinogenesis in the liver. Therefore, Li and colleagues announced that Hepatocellular carcinoma cells modulate endoplasmic reticulum stress by autophagy, which is important for insulin resistance-mediated chemoresistance (100). The study’s findings, supported by experiments that stimulated and hindered autophagy, exhibited that IR-induced elevtaed autophagy remarkably improves chemotherapeutic drug resistance in hepatoma cells. The fact that autophagy regulates endoplasmic reticulum stress in IR-mediated chemoresistance in HCC could be one explanation. Ultimately, autophagy helps hepatocellular carcinoma cells survive chemotherapeutic drug treatment by keeping the ER in homeostasis, suggesting that autophagy’s regulatory function in ER stress contributes to IR-mediated chemoresistance. Moreover, Liu and co-workers in their investigations revealed that osteopontin promotes chemo-resistance in human hepatocellular carcinoma cells via inducing autophagy (101). The findings demonstrated that OPN secretion promoted autophagy by binding to its receptor integrin αvβ3 and maintaining the stability of FoxO3a. Autophagy induced by OPN may enhance the survival of cancer cells, fortify them against chemotherapy, and impart stem-like characteristics (**Figure 3**).

## 5 miRNAs regulate autophagy in hepatocellular carcinoma

Noncoding RNAs (ncRNAs), such as microRNAs (miRNAs), are gaining increasing interest as potential novel therapeutic targets for human disorders (102). MiRNAs are a group of naturally occurring, small noncoding RNAs that control gene expression after the process of transcription. The research of the connection between ncRNAs and autophagy in HCC has gained significant attention in recent years. The significance of miRNAs in physiological and pathological processes, such as tumor development and progression, is becoming increasingly apparent. Plethorus microRNAs participate in the autophagy regulation of HCC (103). Furthermore, in HCC, the expression of several microRNAs is abnormal, and the diverse repercussions that follow from this abnormal expression are numerous (104). Transcription factors, such as NFI-A, PU.1, and C/EBPs, regulate the miR-223 gene, which is located on Xq12. Importantly, miR-233 regulates particular inflammatory responses, thereby exerting a significant impact on the development and maintenance of the immune system (105, 106). Moreover, it improves metastasis and drug resistance in gastric cancer, exacerbates the proliferation of breast cancer cells, and induces carcinogenic influences in gastric cancer (107). It has been revealed that doxorubicin-induced autophagy of HCC cells is facilitated by a decreased rate of miR-223 expression, targeting FOXO3a and so reduces the doxorubicin sensitivity of HCC cells. In contrast, previous studies have demonstrated that doxorubicin treatment for HCC is enhanced by miR-223 overexpression (108). Pihosphatide-3-kinase regulatory subunit 3 (PIK3R3) is distinguished as an oncogene, whereas miR-513b-5p is a miRNA that is downregulated in HCC cells. Autophagy is prevented during the malignant progression of HCC as a result of the impedement of PIK3R3 expression by miR-513b-5p. MiR-513b-5p is thus a possible therapeutic target for HCC (109). In this regard, scientists studied the function of miR-375, a noticeably suppressed miRNA in HCC. They uncovered that miR-375 impeded autophagy by decreasing the levels of Atg7. Consequently, this led to a decrease in the survival of HCC cells in both laboratory cultures and mice in hypoxic conditions. MiR-375 prevented the transformation of LC3-I to LC3-II in low oxygen conditions, thereby obstructing the flow of autophagy. This prevention of mitochondrial autophagy in HCC cells led to a reduction in the elimination of damaged mitochondria, a raise in the release of mitochondrial apoptotic proteins, and finally impaired the survival of HCC cells (110, 111).

miR-101 is observed on chromosomes 1 and 9, severally. It is indispensable in numerous forms of cancer for processes such as invasion, metastasis, drug resistance, angiogenesis, apoptosis, and proliferation (112, 113). It has been found that overexpression of miR-101 impedes autophagy through its regulation of RAB5A, stathmin 1, ATG4D, and other targets. On top of that miR-101 expands apoptosis in HepG2 cells when applied in conjunction with cisplatin, indicating that it extends the sensitivity of HepG2 cells to cisplatin. The opposite takes place when miR-101 expression is insignificant (114, 115). There are currently a limited number of investigations pertaining to miR-4790-3p. MiR-4790-3p expression is reduced significantly in HCC patients treated with a combination of Ku0063794 and everolimus. Conversely, miR-4790-3p targets zinc finger protein 225 (ZNF225) expression, is noticeably raised. By expanding ZNF225 expression, autophagy is prevented when miR-4790-3p is downregulated; consequently, HCC cell survival is dropped (116). Furthermore, Lan and colleagues exhibited that autophagy regulates the expression of miR224 selectively via an autophagosome-mediated degradation mechanism. Additionally, they discovered that off-label use of the antiarrhythmic agent amiodarone effectively inhibited HCC tumorigenesis *in vitro* and *in vivo* via autophagy-mediated miR224 degradation (117). The results of another investigation showed that an increase in cellular autophagy facilitated the inhibition of migration and invasion by glycine decarboxylase overexpression. The impact was diminished through the transfection of miR-30d-5p (118). Additionally, in HCC, autophagy-related genes are targeted by microRNAs to regulate autophagy. Downregulation of miR-30a, which mediates autophagy dependent on Beclin 1 and Atg5 and confers anoikis resistance in HCC cells, was observed in metastatic HCC, based on the research conducted by Xiu-Tao Fu and colleagues (119). Additionally, under hypoxic conditions, the first miRNA with proapoptotic functions, mir-375, can inhibit autophagy and reduce cell viability in HCC cells by binding directly to ATG7 (111).

HCC processes may be inhibited by miR-559 degradation. Cell proliferation and metastasis in numerous varieties of cancer are regulated by Par-3 family cell polarity regulator (PARD3). MiR-559 has the potential to be utilized as a therapeutic target for HCC, as evidenced by research indicating that it inhibits autophagy by suppressing PARD3 expression, thereby inhibiting the growth of HCC (120). Ancient miR-7 (121), is determined to be translated from three genomic loci: 9q21, 19q13, and 15q26 (122). It regulates numerous signaling pathways, including those that promote apoptosis by downregulating the PI3K and MAPK pathways, whereas its primary function is to inhibit tumor growth, survival, and migration (121). Autophagy is accelerated and metastasis and invasion of HCC cells are promoted as a result of the upregulation of ATG5 expression in HCC tissues induced by the downregulation of miR-7 (123). There exists an association between the upregulation of miR-25 in HCC tissues and the clinical stage, lymph node metastasis, and pathological grade. In order to stimulate autophagy and decrease F-box and WD repeat domain containing 7 protein expression, miR-25 increases HCC resistance to sorafenib. Consequently, miR-25 could potentially serve as an innovative therapeutic target for HCC (124). An additional investigation examined the potential autophagy inhibitory effects of miR-26 family members (miR-26a, miR-26b, and miR-26a/b). These inhibitors increased the sensitivity of HCC cells to doxorubicin (Dox) and induced apoptosis by directly impeding the expression of serine/threonine protein kinase ULK1, a crucial autophagy promoter (125). The miR-125b gene, which is located on chromosome 21q21, is an essential constituent of the miR-125 family and is implicated in the commencement and progression of cancer (126). Previous investigations have indicated that oxaliplatin-resistant HCC cells illustrate low rates of miR-125b expression. Conversely, overexpression of miR-125b prevents invasion, proliferation, and epithelial-mesenchymal transition (EMT), proposing that it might expand cell sensitivity to oxaliplatin. Through its mechanistic impediment of EMT and autophagy via downregulation of Eva-1 homolog A, miR-125b effectively declines oxaliplatin resistance in liver cancer patients (127). miR-199a-5p is a member of the miR-199a family and is implicated in driving the development of lung cancer tumors (128). In patients with HCC who are undergoing cisplatin chemotherapy, miR-199a-5p expression has been found to be noticeably lessened. Autophagy is instigated by the downregulation of miR-199a-5p induced by cisplatin via its targeting of ATG7; thus, cisplatin resistance in HCC is expanded (129). miR-193a-3p is a tumor suppressor gene located on chromosome 17 and is implicated in the vast majority of cancer types (130). miR-193a-3p is modulated in HCC by the tumor inhibitor gene mitogen-inducible gene 6 (Mig-6). TGF-2 is a miR-193a-3p target. By positively regulating miR-193a-3p, mig-6 decreases the rate of TGF-2, thereby extending apoptosis and impeding autophagy in HCC (131).

## 6 Therapeutic compounds target autophagy in hepatocellular carcinoma

Flavopereirine triggers autophagy as a means to hinder the advancement of HCC (132). Moreover, resveratrol indicates effectiveness as an anti-cancer drug against HCC and prevents Akt signaling by upregulating the expression of phosphatase and tensin homolog (PTEN), thereby reducing the malignancy of HCC (133). Resveratrol improves the body’s ability to fight against tumors in HCC by declining the population of CD8+ CD122+ Treg cells (134). Furthermore, resveratrol lessens the expression of Gli-1 in the suppression of HCC (135). Resveratrol can enhance the expression of p53 and impede the PI3K/Akt signaling pathway, resulting in the induction of autophagy and the prevention of HCC progression (136). The AMPK/mTOR/p70S6K axis is stimulated by isoqerucetin, which facilitates apoptosis and autophagy in HCC. The anticancer effect of autophagy in HCC is confirmed by the fact that inhibition of autophagy reduces apoptosis by inhibiting capase-3 activation and PARP cleavage and decreasing the Bax/Bcl-2 ratio (137). Dehydrocostuslactone (DCL) and costunolide (CL) were utilized in one experiment as two bioactive components of an extract of sesquiterpene lactones for the treatment of HCC. CL and DCL facilitate the buildup of LC3 and p62 in order to inhibit autophagy and impede the progression of HCC (138). In another investigation, Zou and colleagues reported that autophagy-mediated antitumor activity in human HCC cells is demonstrated by Oroxylin A, a natural monoflavonoid extracted from Scutellariae radix. The activity is dependent on both dose and duration (139). Besides, Berberine, Allicin, Matrine, and Glycyrrhetinic acid are also molecules derived from plants that induce apoptosis and/or autophagy of HCC cells, thereby exerting antitumor effects (140, 141). Moreover, dioscin is a considerably used anticancer drug in the treatment of HCC. Dioscin prevents the process of EMT mediated by TGF-β1 in HCC, hence decreasing the spread of cancer cells (142). Furthermore, dioscin expands the expression of Bax and caspase-3 while declining the rates of Bcl-2 during the process of inducing apoptosis in HCC (143). Dioscin induces apoptosis, autophagy, and DNA damage in HCC cells, resulting in dropped cell growth and metastasis. Dioscin induces autophagy and promotes development of HCC cells by increasing the levels of Beclin-1 and LC-3, and decreasing the rates of p-Akt and p-mTOR (144). A natural bufadienolide derived from toad venom, Arenobufagin, additionally inhibits the PI3K/AKT/mTOR pathway to instigate apoptosis and autophagy in human HCC cells (145). Antibacterial, antifungal, antiviral, anti-inflammatory, and potentially anticancer are some of the biological properties exhibited by -Thujaplicin, a natural derivative of tropolone (146, 147). According to one study, by stimulating autophagy, -Thujaplicin may impede the proliferation of HCC cells (148). Baicalin has primarily been represented to induce apoptosis and cell cycle arrest in tumor cells, thereby exerting its anticancer properties. HCC cells are induced to undergo autophagic cell demise by baicalin (149). An alternative investigation found that the aqueous extract (PB) of P. bistorta (Bistorta officinalis (synonym Persicaria bistorta) stimulated autophagy in HCC, which in turn initiated caspase-dependent apoptosis. Due to its anticancer properties, P. bistorta is utilized in traditional Chinese medicine (150). **Table 1** summarizes the role of autophagy in HCC.

**Table 1:** The function of autophagy in modulation of HCC progression.

Molecular factor	Remark	Reference
TGF-β1	Platelets enhance hepatocellular carcinoma metastasis by promoting epithelial-mesenchymal transition and cancer cell autophagy through the TGF-β1/AMPK/mTOR pathway.	(151)
–	Combining autophagy inhibitors with energy restriction mimetic agents enhances their effectiveness against hepatocellular carcinoma by promoting cancer cell death through apoptosis and cell cycle arrest.	(152)
Nrf2	Sarmentosin induces autophagy and apoptosis in hepatocellular carcinoma cells through Nrf2 activation and mTOR inhibition, effectively suppressing tumor growth *in vivo*.	(153)
AFP	β-Sitosterol inhibits hepatocellular carcinoma progression by targeting the complement C5a receptor 1/AFP axis to activate autophagy and suppress cell proliferation and migration.	(154)
AMPK/mTOR	Ginsenoside Rk1 inhibits hepatocellular carcinoma progression by activating toxic autophagy and apoptosis via the AMPK/mTOR pathway, presenting a promising new treatment strategy for HCC.	(155)
ATG5	Inducing ferroptosis, facilitated by m6A modification of ATG5 mRNA mediated by WTAP and YTHDC2, effectively suppresses hepatocellular carcinoma development, highlighting a promising therapeutic approach.	(156)
UHRF2	UHRF2 enhances autophagy and oncogenic traits in hepatocellular carcinoma (HCC) by interacting with PRDX1 and PARP1, suggesting its potential as a biomarker and therapeutic target for HCC.	(157)
–	Diallyl sulfide (DAS) inhibits the growth of hepatocellular carcinoma cells by disrupting autophagic flux, specifically by blocking autophagosome-lysosome fusion and increasing lysosomal pH, thus enhancing its growth-inhibitory effects when combined with an autophagy inhibitor.	(158)
NDUFS1	Agrimol B inhibits hepatocellular carcinoma growth by initiating autophagy and blocking autophagosome-lysosome fusion, driven by caspase 3-mediated degradation of NDUFS1, which leads to mitochondrial reactive oxygen species accumulation and autophagy arrest, enhancing the efficacy of sorafenib in treating HCC.	(159)
LncRNA XXYLT1-AS2	LncRNA XXYLT1-AS2, highly expressed in HCC plasma, promotes tumor growth by inhibiting autophagy and enhancing proliferation, migration, and invasion of HCC cells, through the degradation of TFEB via the ubiquitin proteasome pathway.	(160)
GPR50	GPR50, significantly upregulated in hepatocellular carcinoma (HCC) patients and the CBRH-7919 cell line, promotes HCC progression by enhancing cell proliferation, migration, and autophagy, mediated through interactions with CCT6A and PGK1, suggesting GPR50 as a potential therapeutic target for HCC.	(161)
ATP1A1	Dysregulated ATP1A1 signaling, influenced by metabolic stress in MASH-related hepatocellular carcinoma (HCC), promotes HCC progression through epigenetic modifications like H3K9 acetylation and tri-methylation, which are linked to decreased autophagy and increased apoptosis when normalized, highlighting ATP1A1 as a potential therapeutic target.	(162)
UBA52	UBA52 promotes hepatocellular carcinoma (HCC) progression by regulating autophagy via EMC6, as evidenced by its association with increased HCC cell proliferation and migration *in vitro* and *in vivo*. Knockdown of UBA52, which induces autophagy and reduces tumor growth and metastasis, suggests its potential as a therapeutic target for HCC.	(163)
NPC1	An eight-gene signature, including NPC1 which is associated with increased immune checkpoint inhibitor sensitivity, promotes hepatocellular carcinoma progression by activating autophagy and enhancing tumor cell proliferation, migration, and invasion.	(164)
AMPK/mTOR	Nicotinamide mononucleotide (NMN) inhibits hepatocellular carcinoma progression by increasing NAD+ levels, enhancing apoptosis, autophagy, and ferroptosis, and activating the AMPK/mTOR pathway, suggesting its potential as a therapeutic agent for HCC.	(165)
NR0B1	NR0B1 is a poor prognostic factor in hepatocellular carcinoma (HCC) that enhances sorafenib resistance by activating autophagy and inhibiting apoptosis, suggesting it as a detrimental influence on HCC treatment outcomes.	(166)
MSDF	9-methanesulfonylmethylene-2,3-dimethoxy-9H-fluorene (MSDF), a novel fluorene derivative, exhibits potent anticancer effects in hepatocellular carcinoma (HCC) by promoting reactive oxygen species (ROS) generation, triggering both extrinsic and intrinsic apoptosis pathways, enhancing autophagy, and reducing the expression of immune checkpoint proteins, positioning it as a promising multitarget drug for HCC treatment and potentially enhancing immunotherapy effectiveness.	(167)
PI3K/AKT/mTOR/HIF-1α	Piezo-CAP, a cold atmospheric plasma technology, effectively suppresses hepatocellular carcinoma by inducing apoptosis and autophagy through targeting redox balance, glycolysis, and the PI3K/AKT/mTOR/HIF-1α signaling pathways.	(168)
TMX2	TMX2 enhances hepatocellular carcinoma (HCC) cell viability by promoting autophagy and mitophagy, is upregulated in HCC tissues, associated with poor prognosis, and potentially serves as a therapeutic target.	(169)
CDK5	CDK5 deficiency in hepatocellular carcinoma (HCC) cells upregulates PD-L1 through decreased phosphorylation and reduced chaperone-mediated autophagy, enhancing response to anti-PD-1 immunotherapy and improving survival in HCC-bearing mice.	(170)
GDF11	GDF11 acts as a tumor suppressor in hepatocellular carcinoma (HCC) by inhibiting cell proliferation, migration, and angiogenesis, while promoting apoptosis and autophagy via inactivation of the mTORC1 signaling pathway.	(171)
–	Icaritin suppresses hepatocellular carcinoma growth by inducing mitophagy and apoptosis, with inhibition of mitophagy enhancing its anticancer efficacy, suggesting a novel strategy for improving HCC treatment outcomes.	(172)
mTOR/STAT3	Trillin inhibits autophagy and promotes apoptosis in hepatocellular carcinoma cells through activation of the mTOR/STAT3 signaling pathway, suggesting its potential as a therapeutic agent for HCC.	(173)
SLC5A7 and p53	Choline suppresses hepatocellular carcinoma progression by inhibiting autophagy via upregulation of SLC5A7 and p53, and enhances the efficacy of sorafenib in treating HCC.	(174)
IL7 and MAL2	IL7 and MAL2 promote Sorafenib resistance in hepatocellular carcinoma by enhancing JAK/STAT and PI3K/AKT signaling, while autophagy-inducing stapled peptides counteract this resistance by degrading resistance-related proteins and synergizing with Sorafenib.	(175)
PI3K/Akt	Physalin A promotes apoptosis and autophagy in hepatocellular carcinoma cells by inhibiting the PI3K/Akt signaling pathway, effectively reducing tumor growth *in vivo*.	(176)
SIRT1	SIRT1 plays a critical role in promoting autophagy and modulating NF-κB signaling in both sorafenib-resistant and parental hepatocellular carcinoma cells, highlighting its potential as a therapeutic target in HCC treatment.	(177)
SRXN1	SRXN1 enhances hepatocellular carcinoma progression by modulating lysosome biogenesis and autophagic flux, with its inhibition showing synergistic antitumor effects with sorafenib by increasing ROS levels, positioning it as a potential therapeutic target for HCC.	(178)
CIP2A/p-AKT/c-Myc	JXE-23, a pimarane-type diterpene, exhibits potent anti-cancer effects in HepG2 liver cancer cells by inducing G2/M cell cycle arrest and protective autophagy, and modulating the CIP2A/p-AKT/c-Myc signaling pathway, suggesting its potential as a lead compound in anti-cancer drug development.	(179)
AMPK/AKT/mTOR	The baculovirus-mediated endostatin and angiostatin fusion protein (BDS-hEA) inhibits hepatocellular carcinoma growth and angiogenesis by inducing autophagy through the AMPK/AKT/mTOR signaling pathway, enhancing its therapeutic efficacy.	(180)
–	Amplifying autophagy in residual tumor cells using targeted nanoparticles enhances immunogenic cell death and anti-tumor immunity, presenting a novel strategy to combat tumor recurrence and resistance to anti-PD-1/PDL1 therapy post-incomplete radiofrequency ablation.	(181)
–	A bioactive fraction from pine needle extract containing pinocembrin, chrysin, and tiliroside significantly enhances apoptosis and reduces autophagy in hepatocellular carcinoma cells, suggesting its potential as an effective complementary anticancer therapy.	(182)
AGC1	AGC1 plays a crucial role in reprogramming glutamine metabolism and maintaining energy supply in hepatocellular carcinoma cells, making it a potential target to enhance the efficacy of therapies aimed at inhibiting glutamine dependency in HCC.	(183)
ClpP	ONC206 inhibits hepatocellular carcinoma growth by inducing apoptosis and cytoprotective autophagy mediated by ClpP-induced mitochondrial dysfunction, with enhanced effects upon autophagy blockade.	(184)
STIM1	STIM1 promotes autophagy and epithelial-mesenchymal transition in hepatocellular carcinoma by interacting with LC3B through its SAM domain, offering a potential target to inhibit HCC metastasis.	(185)

## 7 Ferroptosis machinery

**Figure 4:**
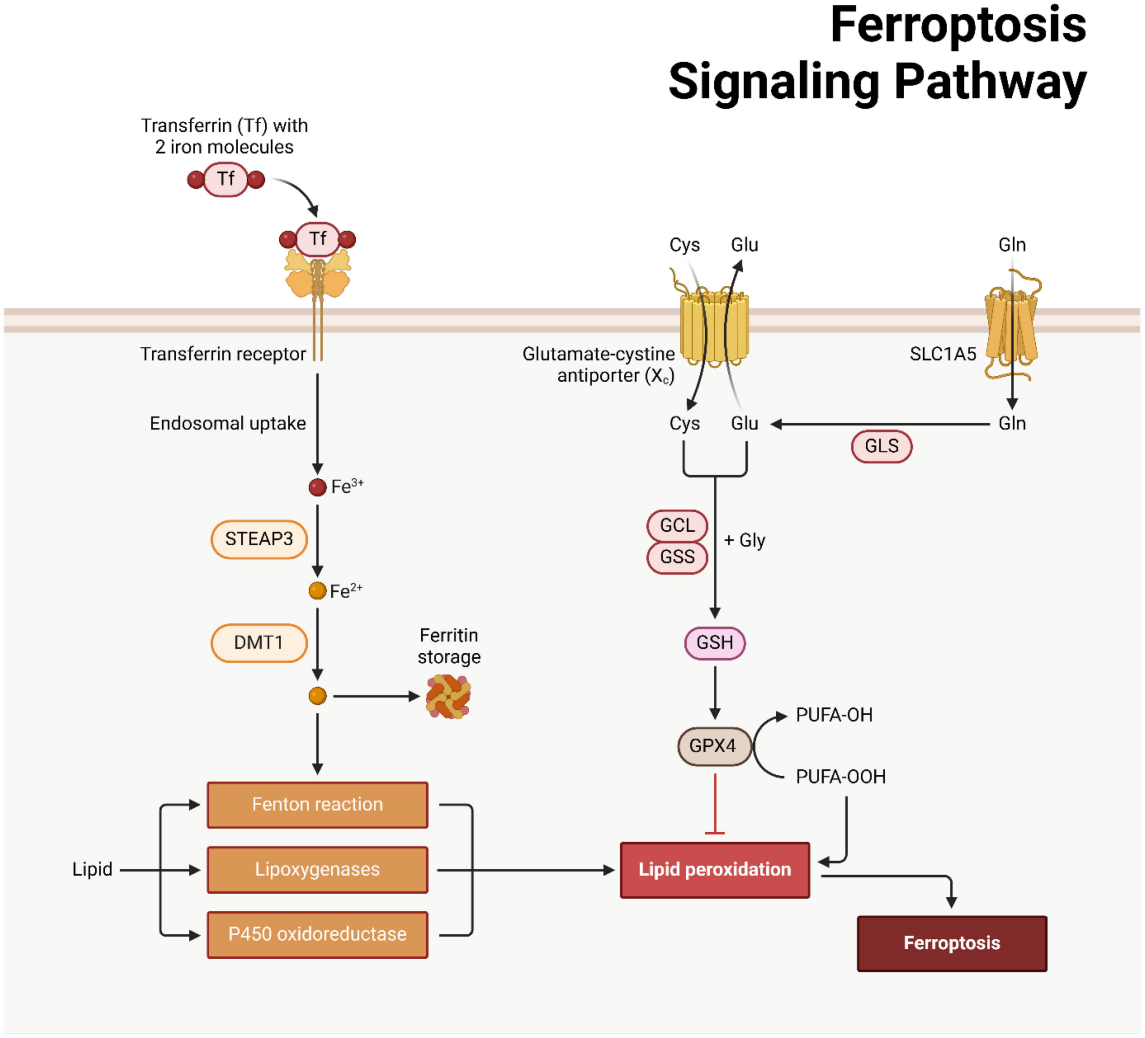
An overview of ferroptosis machinery.

The regulated cell death phenomenon known as ferroptosis was initially documented by Dixon and colleagues in 2012 (186, 187). In 2008, Yang and Stockwell were the first to identify compounds capable of causing death to cells carrying mutant subtypes of ras sarcoma (RAS) (188). By means of phospholipid peroxidation, where iron metabolic products, polyunsaturated fatty acids, and reactive oxygen species (ROS) interact, an erythrostatin is capable of inducing a nonapoptotic form of cell death (187). System xc− and glutathione peroxidase 4 are critical pathways in the initiation, execution, and regulation of ferroptosis. Additionally, lipid and iron peroxidation are crucial to ferroptosis. A concise overview is also provided of regulatory factors such as nuclear factor erythroid 2-related factor 2 (NRF2) and ferroptosis suppressor protein 1 (FSP1). An amino acid antiporter, System xc∓ plays a role in the translocation of L-cystine from outside the cell to within the cell via the plasma membrane. belonging to the HAT family (189). It is a heterodimer composed of SLC7A11 and SLC3A2 connected by a disulfide bond. Critical for the survival of mammalian cells, GPX4 is a restricted selenoprotein that can mediate the reduction of lipid peroxides (LPOs) in a complex cell membrane environment. By impeding GPX4, lipid peroxidation can be increased and ferroptosis, the origin of LPOs in healthy cells, can be induced (190–192). One of the most lethal signals of ferroptosis, GPX4 serves as the principal regulatory factor in the process. Additionally, glutathione (GSH) and cysteine, which function as fundamental auxiliary factors for GPX4, exert control over it (192, 193). By influencing GPX4 activity, System xc∢ can modulate intracellular cysteine levels and thus control ferroptosis. SLC7A11 is concurrently regulated by numerous genes, some of which function as tumor suppressor genes with a negative regulatory function (194–196). The expression of system xc− and GPX4 can be inhibited, respectively, by erastin and RSL3 (**Figure 4**) (197, 198).

Ferroptosis and cellular metabolism have a complex relationship (199, 200), with lipid peroxidation serving as a crucial driver (187). The initiation, propagation, and termination phases of this process are intricately interconnected (201, 202). In the event that GPX4 is unable to eradicate redundant LPOs, ferrous iron converts them to an alkoxyl radical (LO³) (201). As a consequence, the lipid peroxidation process fails to progress to its ultimate termination phase, which facilitates the buildup of LPOs, including malondialdehyde (MDA), which may induce cascades of cellular death or toxicity (201, 203). Iron-containing LOXs, which are nonheme enzymatic protein effectors, are of paramount importance in lipid peroxidation during ferroptosis. A LOX knockout has the ability to withstand ferroptosis mediated by erastin (204, 205). The p53/SLC7A11/12-LOX pathway is additionally implicated in the ferroptosis-induced production of LPO (203, 206). In ferroptosis, lipid peroxidation is metabolized via a series of interconnected cascades governed by the equilibrium when it comes to the antioxidant and oxidation systems, l Methods for removing lipids from cells, repairing membranes, and autophagy (207). The role of GPX4 in additional RCD processes provide credence to the idea that lipid peroxidation occurs at the “intersection” of RCD (196, 207, 208). Enhanced release, increased absorption, or restricted intracellular iron efflux are factors that can induce the accumulation of intracellular iron, which can facilitate ferroptosis via multiple pathways (209). The Fenton reaction, a non-enzymatic chain that increases PLOOH production, a critical step in ferroptosis (210), indicates that this process is iron-dependent. Iron is additionally indispensable for specific enzymes that are engaged in ferroptosis, including Cytochrome P450 Reductase (POR) and Phospholipid peroxidation metabolism-related LOX (211). Ferroptosis may be influenced by metabolic regulators associated with iron metabolism, encompassing iron uptake, storage, transport, or degradation. Iron transportation, storage, and release are primarily regulated via the iron regulatory proteins (IRPs)-iron response element (IRE) in mRNA interaction (212, 213). IRE-binding protein 2 (IRE-BP2) is a pivotal element in the process of ferroptosis induction (214). Transferrin (TF) transports iron to various tissues and organs by binding to iron in the circulation. Ferroptosis is mediated by the transferrin receptor (TFR), which is also accountable for the cellular uptake of iron-loaded TF (214). By enhancing TFR function, oncogenic RAS can increase cellular iron stores, thereby increasing susceptibility to ferroptosis induced by erastin (188). Ferritin degradation can be achieved through the mechanism of selective autophagy, which is facilitated by nuclear receptor coactivator 4 (NCOA4) (215). Additional iron metabolism-related proteins or regulators, including prominin-2 (PROM2) and heme oxygenase 1 (HO1), may exert an influence on ferroptosis (216, 217).

## 8 Ferroptosis in hepatocellular carcinoma progression

Because it modifies the cancer cells’ biological characteristics, ferroptosis controls HCC. like cell death, migration, and proliferation, based on an increasing amount of research investigating the link between HCC and ferroptosis (218). An example of a protein that prevents cell proliferation in HCC patients is protocadherin beta 14 (CDHB14). This protein improves ferroptosis by blocking the interaction between p65 and SLC7A11. The downregulation of CDHB14 in HCC patients suggests that it may play a tumor-suppressing role in HCC (219). Ferroptosis and tumorigenesis, however, are linked to a number of the proteins that are overexpressed in HCC. Based on the consequences of Zhang and colleagues, the zinc-finger protein 498 (ZNF498) expands hepatocarcinogenesis and progression while inhibiting p53-triggered apoptosis and ferroptosis (220). ZNF498 is signifying a high clinical grade and a poor prognosis in HCC patients. An independent risk factor associated with the development of hepatocellular carcinoma (HCC) has been identified as glucose-6-phosphate dehydrogenase (G6PD). This enzyme not only inhibits cytochrome P450 oxidoreductase (POR), but also promotes tumor invasion, metastasis, and growth, while decreasing ferroptosis (221). Moreover, Guo and colleagues exhibited that Hepatocellular carcinoma progression is accelerated and prognosis is poor when RPLP2 is highly expressed because it inhibits ferroptosis (222). The study revealed that elevated RPLP2 rates are linked to advanced clinicopathologic features and poor prognosis in HCC patients. The study also found that decreased RPLP2 DNA methylation rates correlate with patient outcomes. High RPLP2 expression is linked to unfavorable immune infiltration and positively associates with ferroptosis suppressor GPX4, potentially accelerating ferroptosis to suppress HCC tumor progression (222). Furthermore, in another research, Lyu and co-workers indicated that through the circ0097009/miR-1261/SLC7A11 axis, ferroptosis contributes to the progression of hepatocellular carcinoma (223). The results illustrated that Circ0097009 expression rised in HCC tissues and cell lines, while knockdown lessened invasion and proliferation. It connects directly to miR-1261 and modulates each other’s expression via miR-1261 sponging. Circ0097009 controls SLC7A11 expression in HCC, an important regulator of cancer cell ferroptosis, acting as a competing endogenous RNA (223). Besides, Bi and collaborators investigated the role of METTL9-SLC7A11 axis in hepatocellular carcinoma and revealed that METTL9-SLC7A11 axis expands HCC progression by preventing ferroptosis (224). Scientists found that inhibiting METTL9 expression decreased levels of SLC7A11, an important regulator of ferroptosis. This, in turn, increased ferroptosis in HCC cells, which slowed the development of HCC. Also, we demonstrated that METTL9 targeting might considerably limit the growth of HCC PDX (224). Induction of ferroptosis may be utilized as a therapeutic approach for HCC, according to these results. Potentially, the treatment of HCC could involve the targeting of ferroptosis suppressors. Shan and collaborators reported that inhibiting the NRF2 signaling pathway does not promote ferroptosis but does impede the growth and progression of HCC. However, CEP290 suppression inhibits ferroptosis (225). Similarly, the RNA-binding protein ENO1, also known as alpha-enolase, inhibits the operation of mitochondrial iron-induced ferroptosis and iron regulatory protein 1 (IRP1), suggesting that it may serve as a fundamental target for therapeutic interventions targeting HCC (226). Resistance mechanisms against anticancer therapies may be established by HCC under hypoxic conditions. SLC7A11 degradation, YTHDF induces this process by attaching to N6-methyladenosine RNA. obstructs ferroptosis and consequently diminishes the effectiveness of HCC interventional embolization (227). A decrease in methyltransferase-like 14 (METTL14) due to hypoxia inhibits ferroptosis. Idh2 knockdown, an enzyme of which NADPH is produced, reduces mitochondrial GSH and thereby increases erastin-induced ferroptosis in hepatocellular carcinoma cells. thereby impeding the progression of HCC, according to another investigation that was profoundly impressed (228). It is necessary to further study the precise function and mechanism of ferroptosis, which is represented to be critical in the initiation and progression of HCC in the aforementioned investigations. As an additional mechanism, ferroptosis aids in the angiogenesis of HCC. In cultured human HCC cells, the miR-17-92 cluster has been linked to an increase in cell proliferation, colony formation, and invasiveness (229). by inhibiting the expression of ACSL4 (230). This mechanism of action aids in the promotion of tumor angiogenesis in HCC and shields endothelial cells from the ferroptosis caused by erastin as an oncogenic miRNA cluster.

In another investigation, Zhang and colleagues was studied the function of mitochondrial translocator protein (TSPO) in controlling ferroptosis and antitumor immunity. They indicated that by inhibiting ferroptosis and immune evasion, mitochondrial TSPO promotes the progression of hepatocellular carcinoma (231). The results showed that TSPO, a protein found in HCC, is expressed and linked to insignificant prognosis. It expands HCC cell growth, migration, and invasion, hinders ferroptosis, and upregulates PD-L1 expression. It also interacts with P62, hindering autophagy and proteasomal degradation. In a mouse model, TSPO inhibitor PK11195 combined with anti-PD-1 antibody indicated a synergistic anti-tumor impact. Research conducted by Yang and colleagues illustrated that the activation of the Nrf2/HO-1/GPX4 axis suppresses the progression of hepatocellular carcinoma through polyphyllin I induced ferroptosis (232). The consequences indicated that PPI prevents HCC cell proliferation, invasion, and metastasis by enhancing reactive oxygen species, improving Fe2+ accumulation, depleting GSH, and suppressing xCT and GPX4 expression. This induces ferroptosis, which is connected to PPI binding to Nrf2, HO-1, and GPX4 proteins, modulating the Nrf2/HO-1/GPX4 antioxidant axis. Ferrostatin-1 mitigates this disruption. *In vivo*, PPI impedes Nrf2/HO-1/GPX4 axis-induced ferroptosis, hindering HCC progression.

## 9 Ferroptosis in the regulation of hepatocellular carcinoma chemoresistance

The resistance of HCC patients to drugs considerably impairs the effectiveness of chemotherapy. Inducing ferroptosis is a crucial chemotherapeutic function of sorafenib in the treatment of HCC. The susceptibility of HCC cells to sorafenib can be influenced by a multitude of ferroptosis regulators. In their study, Feng and colleagues provided evidence that ACSL4, an enzyme that activates ferroptosis, can amplifie the ferroptosis induced by sorafenib and effectively forecasts the sensitivity of HCC to sorafenib (233). Moreover, Sun and collaborators showed that QSOX1, an enzyme involved in quiescin hydrate oxidation, makes HCC cells more vulnerable to oxidative stress and expands the ferroptosis induced by sorafenib through the inhibition of NRF2. These results suggest that QSOX1 may serve as an underlying therapeutic target for HCC (234). By inhibiting ferroptosis in HCC, blocking the activity of the N6-methyladenosine reader insulin-like growth factor 2 mRNA-binding protein 3 (IGF2BP3) renders NRF2 mRNA unstable and thus circumvents sorafenib resistance (235). Zeta-1 glutathione S-transferase, an enzyme that metabolizes phenylalanine, also inhibits the NRF2/GPX4 axis in sorafenib-resistant HCC cells. This leads to an increase in lipid peroxidation and ferroptosis, which in turn increases the sensitivity of HCC cells to sorafenib (236). Furthermore, phosphoseryl-tRNA kinase (PSTK) suppression promotes ferroptosis. and enhances the therapeutic impact of sorafenib in hepatocellular carcinoma (HCC) by preventing the activity of GPX4 and additional disruption of cellular glutathione levels (237). In addition, accelerated secretion rates of the cysteine-rich, acidic protein (SPARC) contribute to the cytotoxic influences of sorafenib in HCC by raising reactive oxygen species (ROS) and promoting ferroptosis (238).

Furthermore, Gao and colleagues revealed that by inhibiting ferroptosis, YAP/TAZ and ATF4 cause hepatocellular cancer to resist Sorafenib (239). The transcription factors YAP/TAZ were recognized as crucial catalysts of Sorafenib resistance in HCC through the prevention of Sorafenib-induced ferroptosis in this investigation, which utilized a combination of shRNA-mediated synthetic lethality screening and transcriptomic analysis. SLC7A11, a crucial transporter that regulates intracellular glutathione homeostasis, is mechanistically induced by YAP/TAZ in a manner dependent on TEAD. This mechanism allows HCC cells to surmount the ferroptosis induced by Sorafenib. Simultaneously, YAP/TAZ maintain the function of ATF4, facilitating the induction of SLC7A11 expression and ensuring its nuclear localization and protein stability. Furthermore, Huang and colleagues illustrated that by blocking SLC7A11-induced ferroptosis in hepatocellular carcinoma, ABCC5 aids in the development of sorafenib resistance (240). The results showed that ABCC5 expression is induced in sorafenib-resistant HCC cells, resultig in poor clinical prognoses. Deregulating ABCC5 expression can reduce sorafenib’s resistance to HCC cells. The PI3K/AKT/NRF2 axis is essential for triggering ABCC5 expression. ABCC5 increases GSH and attenuates lipid peroxidation, inhibiting ferroptosis. Inhibiting ABCC5 enhances sorafenib’s anti-cancer activity *in vitro* and *in vivo*.

Nevertheless, it has been revealed that a number of negative ferroptosis regulators has the ability to imake HCC more resistant to sorafenib. For example, ABCC5, It belongs to the subfamily C of ATP binding cassettes, is noticeably expressed in HCC cells that are resistant to sorafenib. This protein expands Glutathione (GSH) and stabilizes SLC7A11, which decreases lipid peroxidation. This, in turn, leads to the suppression of ferroptosis and induces resistance to sorafenib (240). Similarly, DAZAP1, azoospermia associated protein 1 is connected with a protein that has a negative impact on the clinical prognosis of HCC., has the ability to prevent ferroptosis through connecting to SLC7A11 messenger RNA. This, in turn, reduces the susceptibility of HCC cells to sorafenib (241). The research conducted by Gao L. and colleagues (2021) shed light on the fact that YAP/TAZ promotes SLC7A11 expression by interacting with activating transcription factor 4 (242). This, in turn, makes it possible for HCC cells to escape the ferroptosis that is induced by sorafenib. Based on these findings, it appears that ferroptosis-related regulators have the ability to target SLC7A11 in order to minimize sorafenib resistance associated with HCC. A higher level of the enzyme branched-chain aminotransferase 2 (BCAT2) is also found in tissues affected by HCC. This enzyme prevents cancer cells from dying from sorafenib-induced ferroptosis by raising glutamate levels. However, reducing system Xc-activity can decrease this impact. as stated by Wang and colleagues (243). It has been observed that helicosis causes an overexpression of the sigma-1 receptor (S1R). that has been treated with sorafenib. Ferroptosis results from a decrease in this receptor, which inhibits GPX4 synthesis. including increased susceptibility to sorafenib, lipid peroxidation, and iron metabolism (244). Similarly, the inhibition of the leukemia inhibitory factor receptor (LIFR) is responsible for initiating the process of hepatocarcinogenesis. This inhibition also imparts resistance to sorafenib-induced ferroptosis on hepatocellular carcinoma cells by boosting the activity of the NF-κB/LCN2 (lipocalin 2) pathway (245). The elimination of HCC cell resistance to sorafenib could therefore be accomplished by targeting ferroptosis-suppressors.

## 10 Non-coding RNAs regulate ferroptosis in hepatocellular carcinoma

Research has shown that ncRNAs linked to ferroptosis play a role in the beginning, progression, outcome, and resistance to drugs in HCC. Although ferroptosis is activated by some tumor-suppressing ncRNAs, while others target components involved in ferroptosis. In this regard, the research conducted by Zhang and colleagues provided confirmation that the long non-coding RNA HEPFAL is essential for facilitating ferroptosis through the downregulation of SLC7A11 expression. This discovery underscores the potential therapeutic usefulness of lncRNA HEPFAL in the treatment of HCC. It is noteworthy that lncRNA NEAT1 represents upregulation in erastin-exposed HCC cells. It serves as a surrogate for miR-362-3p, thereby increasing ROS generation and decreasing NADPH and GSH levels. A faster rate of ferroptosis in HCC cells is the result of increasing myoinositol oxygenase (MIOX) expression (220). Apoptosis and growth inhibition of HCC cells are both aided by ketamine. Through the lncRNA PVT1/miR-214-3p/GPX4 axis, ketamine inhibits HCC cell malignant behaviors and causes ferroptosis (234). The negative regulator of ferroptosis is ATF4, and miR-214 has the potential to function as an ATF4 inhibitor. In their study, Bai and collaborators revealed that the upregulation of miR-214 leads to elevated concentrations of Fe2+, reactive oxygen species, and malondialdehyde through the inhibition of ATF4, this allows erastin-mediated ferroptosis to target HCC cells (246). Qi and colleagues discovered lncRNA GABPB1-AS1, which further decreases HCC cells’ antioxidant potential by inhibiting the production of PRDX5 (peroxiredoxin 5) peroxidase., thereby promoting erastin-induced ferroptosis, along with the finding that GABPB1 is both highly expressed and linked to a poor prognosis in HCC (247). The results of this study suggest that ncRNAs have the ability to stimulate ferroptosis, which could represent a viable therapeutic approach for HCC.

Furthermore, LncRNAs have a key role in the development and advancement of HCC by inhibiting ferroptosis. A predictive model for the diagnosis, therapy, and prognosis of HCC can be constructed by examining lncRNAs linked to ferroptosis. By classifying tumors, the prognostic model developed by Xie and colleagues using the ferroptosis-related lncRNA signature may enhance the ability to predict the survival of HCC (248). The lncRNAs associated with ferroptosis are crucial in the context of hepatocellular carcinoma (HCC) immunosuppression, genomic instability, and therapeutic responsiveness. Their identification could be advantageous in the pursuit of personalized prognosis and treatment strategies for HCC patients (249, 250). A ferroptosis-related long non-coding RNA (lncRNA) model was developed by Wang and collaborators to effectively forecast the prognosis of hepatocellular carcinoma (HCC), a factor that is correlated with tumor grade and macrophage and fibroblast infiltration (251). Additional investigation unveiled that these prognostic models might exert control over the immune microenvironment of HCC through modulation of immune-related pathways, including IL-2 STAT5 and TNF-α/NF-κB. This, in turn, influences the differentiation of immune infiltration (252, 253). Additional prediction models have identified lncRNAs associated with ferroptosis that are also involved in tumor immunotherapeutic efficacy, chemotherapeutic, and tumor microenvironment modification in HCC (254, 255). In addition, seventeen lncRNAs associated with ferroptosis have been identified as a risk and prognostic model with the purpose of enhancing the accuracy of clinical treatment and therapeutic decisions pertaining to HCC (256). Significantly, MALAT1 (metastasis-associated lung adenocarcinoma transcript 1), a critical lncRNA implicated in the progression of HCC, has the capacity to influence cellular proliferation, apoptosis, and migration (257). Overexpression of the HCC prognostic gene MALAT1 is related with a worse prognosis and a lower overall survival rate in patients with HCC (258). However, additional research is required to determine how MALAT1 influences HCC biological activity via its interactions with signaling pathways involved in ferroptosis. Nevertheless, a number of studies have demonstrated that ncRNAs stimulate ferroptosis, thereby playing an oncogenic function in HCC. On sorafenib-resistant HCC cells, Lu et al. investigated the chemotherapeutic implications of miR-23a-3p. In patients who did not respond to sorafenib, miR-23a-3p was found to be overexpressed, indicating a poor prognosis in HCC. Through the promotion of ferroptosis, silencing miR-23a-3p expedited sorafenib’s sensitivity to HCC. By reducing iron buildup and lipid peroxidation, MiR-23a-3p mediates sorafenib resistance through suppressing ACSL4. according to additional molecular research (259). Moreover, Guan and colleagues indicated that the inhibition of proliferation and metastasis in cancer cells occurs when lncRNA HULC is depleted, via the miR-3200-5p/ATF4 Axis, which enhances ferroptosis and oxidative stress in HCC cells (260). An identical inquiry was conducted by Xu and co-workers regarding the impact of miR-541-3p on the progression of HCC cells via circIL4R-mediated effects. CirculIL4R expression is significantly elevated in HCC tissues relative to their corresponding normal samples, according to their research. By blocking GPX4 expression and miR-541-3p sponging, circIL4R promotes tumors and inhibits ferroptosis in HCC cells through the miR-541-3p/GPX4 axis (261). As a result, Reducing circIL4R activity speeds up the ferroptosis of HCC cells and stops oncogenesis in its tracks. A novel therapeutic target for HCC could be the inhibition of ferroptosis-suppressing ncRNAs, according to these findings.

## 11 Therapeutic compounds target ferroptosis in hepatocellular carcinoma

Traditional Chinese Medicine (TCM) has been shown to be effective in treating HCC, with herbal compounds like Scutellaria barbata, ellagitannin, ardipusilloside-I, Annona squamosa seeds, Panax, and gypenoside showing preventive effects (262–266). TCM compounds can improve clinical symptoms, quality of life, and prolong HCC patient survival (267). In the respect, Dai and co-workers exhibited that the Scutellaria barbata inhibits the tumorigenicity of hepatocellular carcinoma by causing the cells to undergo ferroptosis (256). The findings demonstrated that S. barbata inhibited the proliferation of HCC cells by promoting ferroptosis. The investigation into the molecular mechanism by which S. barbata stimulated ferroptosis in HCC cells revealed that ferroptosis may be induced via iron perioxidation promotion and lipid reactive oxygen species (ROS) metabolism. S. barbata further impeded the tumorigenicity of HCCs *in vivo* through the induction of ferroptosis in HCC cells. Proliferation, growth, and the generation of reactive oxygen species (ROS) are all dependent on iron. Including proteins such as transferrin receptor, ferritin, and ferroportin, its metabolism consists of absorption, storage, and export. Iron is an essential nutrient for cancer cells (268). Thus, Lin and colleagues indicated that human hepatocellular carcinoma cells driven by Saponin Formosanin C to engage in ferritinophagy and ferroptosis (269). The results showed that ferritinophagy is found to be a significant factor in ferroptosis-induced cell death in HepG2 cells with elevated NCOA4 expression and decreased FTH1 levels; this suggests that ferritinophagy may have chemotherapeutic potential against apoptosis-resistant HCC in which NCOA4 expression is elevated.

In another research, Xu and colleagues demonstrated that Tiliroside makes hepatocellular cancer more sensitive to sorafenib by targeting TBK1, which induces ferroptosis (270). The consequences showed that Tiliroside significantly enhanced sorafenib’s anti-HCC activity without side effects, and when combined with sorafenib, it induced synergistic effects against HCC. Tiliroside inhibited TBK1’s enzymatic activity, promoting Keap1-mediated Nrf2 ubiquitination and degradation. *In vivo*, it inhibited the growth of HepG2 tumors in both subcutaneous and orthotopic xenograft tumor models of HCC. The retinoblastoma protein, a key gene transcription regulator, is frequently lost in HCC due to various mechanisms, including loss of heterozygosity, epigenetic alterations, CDK activation, increased ubiquitinylation, and protein turnover, though its impact on HCC response to sorafenib remains unknown (271–276). Thus, Louandre and coleagues demnstrated that in human hepatocellular carcinoma cells, the retinoblastoma (Rb) protein controls the ferroptosis that sorafenib induces (277). The study found that HCC cells with reduced Rb levels show a two- to threefold increase in cell death induction when exposed to sorafenib. In Balb/c nude mice, sorafenib treatment led to complete tumor regression in 50% of treated animals. The Rb-negative status of HCC cells promotes ferroptosis, a form of oxidative necrosis, which is a key role of Rb in HCC cell response and ferroptosis regulation. Another investigation conducted by Suzuki and colleagues indicated that in hepatocellular carcinoma, GLS2 inhibits tumor growth and controls the process of ferroptosis (278). The study revealed that deficiency in GLS2 can lead to resistance to ferroptosis in human cancer cells, cells from Gls2 knockout mice,. GLS2 increases lipid reactive oxygen species production in order to enhance ferroptosis, by changing glutamate into α-ketoglutarate. By expressing a catalytically inactive version of GLS2 or by preventing ferroptosis, the effect of ectopic expression of WT GLS2 on tumor size in a human hepatic adenocarcinoma model was neutralized. GLS2-mediated regulation of ferroptosis is also involved in human tumor suppression.

**Figure 5:**
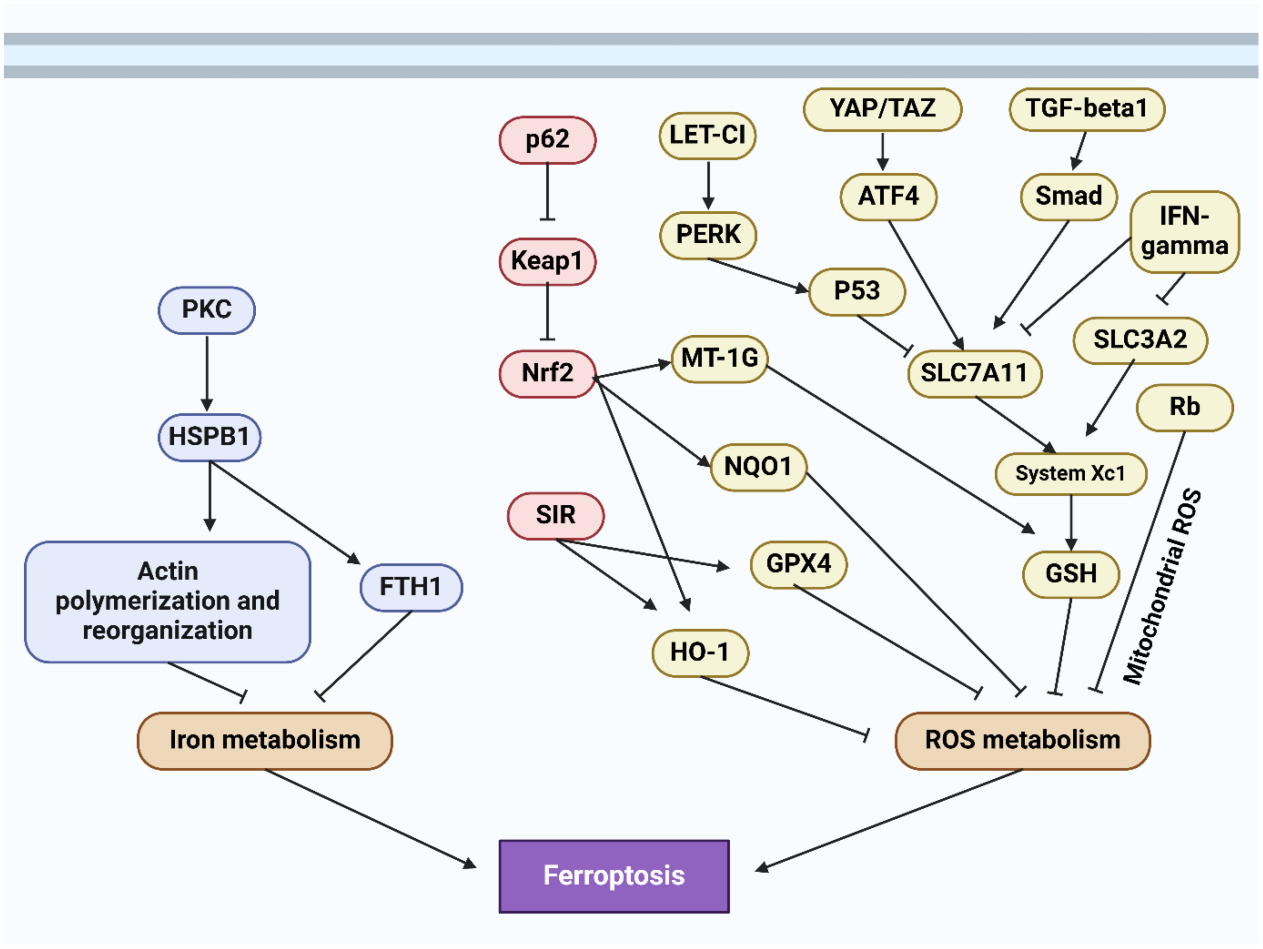
The schematic representation of ferroptosis in hepatocellular carcinoma (285).

Solanum melongena’s major glycoalkaloids, solamargine and solasonine, have potential as anticancer drugs with mild side effects (279, 280). Solasonine has been found to inhibit human colorectal cancer cell progression and lung carcinoma cell invasiveness (281, 282), but its efficacy for treating HCC remains unclear. Therefore, Jin and co-workers indicated that Solasonine enhances hepatoma carcinoma cell ferroptosis by destroying the glutathione redox system in a glutathione peroxidase 4-induced manner (283). Solasonine significantly inhibited the proliferation of HepG2 and HepRG cells, suppressing tumor volume and weight in a mouse xenograft model. Metabolomics analysis revealed that solasonine’s effects on glutathione metabolism, including GPX4 and GSS, were responsible for preventing ferroptosis, a key factor in cell death. Solasonine increased lipid ROS levels in HepG2 cells by suppressing GPX4 and GSS, but using a ferroptosis inhibitor reversed solasonine-induced ROS production and cell apoptosis. Furthermore, Mei and colleagues illustrated that the Aggressiveness of Hepatocellular Carcinoma Cells *in Vitro* Is Inhibited by Rhamnazin via Glutathione Peroxidase 4-Dependent Ferroptosis (284). It was discovered by researchers that rhamnazin inhibits HCC cell proliferation and invasion, with ferroptosis playing a role in this influence. HCC cells were recognized as having elevated concentrations of reactive oxygen species, lipid peroxidation, and iron content. Overexpression of GPX4 inhibited the rhamnazin-induced ferroptotic cell death in which it was discovered to be implicated. **Figure 5** provides an overview of ferroptosis in HCC. **Table 2** summarizes the role of ferroptosis in HCC progression.

**Table 2:** The regulation of ferroptosis during HCC progression.

Molecular factor	Remark	Reference
USP8	Inhibition of USP8, by stabilizing OGT and impacting cystine uptake via SLC7A11, effectively suppresses hepatocellular carcinoma progression and induces ferroptosis, suggesting its potential as a therapeutic target for HCC treatment.	(286)
lncRNA EPS15-AS1	lncRNA EPS15-AS1 induces ferroptosis in liver hepatocellular carcinoma by downregulating EPS15 and AKR1B1, disrupting redox balance and inhibiting cell invasiveness, highlighting its potential as a therapeutic target and diagnostic biomarker for LIHC.	(287)
SLC7A11 and GPX4	Gallic acid induces ferroptosis in hepatocellular carcinoma cells by inhibiting SLC7A11 and GPX4 expression and blocking the Wnt/β-catenin pathway, highlighting its potential as a therapeutic agent for HCC.	(288)
CENPA/STMN1	The prognostic risk model based on eight TF-ferrGene regulatory network-related genes, particularly highlighting the CENPA/STMN1 network, effectively predicts HCC patient outcomes by modulating ferroptosis and influencing malignant phenotypes through transcriptional regulation.	(289)
HNRNPL and S100A9	HNRNPL and S100A9 are overexpressed in hepatocellular carcinoma (HCC) and promote ferroptosis resistance; silencing HNRNPL enhances ferroptosis by reducing cellular antioxidants and increasing oxidative stress markers, while S100A9 overexpression or ferroptosis inhibition reverses these effects, suggesting a targetable mechanism for enhancing ferroptosis in HCC therapy.	(290)
ATF3	Brusatol (BRU) inhibits hepatocellular carcinoma (HCC) growth by inducing ATF3-mediated ferroptosis, highlighting its potential as an effective therapeutic agent for HCC.	(291)
ATF3	Saikosaponin A (SsA) induces ferroptosis in hepatocellular carcinoma (HCC) cells by inhibiting SLC7A11 and activating ATF3 through endoplasmic reticulum stress, highlighting its potential as a therapeutic ferroptosis inducer in HCC treatment.	(292)
ATF4	Dihydroartemisinin (DHA) induces ferroptosis in hepatocellular carcinoma (HCC) by inhibiting ATF4, thus decreasing SLC7A11 expression and enhancing lipid peroxidation, also improving chemosensitivity to sorafenib, offering a novel therapeutic approach for HCC.	(293)
DDX5	DDX5 sensitizes hepatocellular carcinoma (HCC) to sorafenib by inhibiting Wnt/β-catenin signaling and inducing ferroptosis, offering a promising therapeutic strategy when overexpressed in combination with multi-tyrosine kinase inhibitors (mTKIs).	(294)
PIAS3	The study identifies PIAS3 as a key promoter of ferroptosis in hepatocellular carcinoma (HCC) by regulating TXNIP through TGF-β signaling, suggesting a novel therapeutic target for enhancing ferroptotic sensitivity in HCC treatment.	(295)
NeuroD1	NeuroD1 promotes resistance to ferroptosis in hepatocellular carcinoma (HCC) by upregulating GPX4, enhancing cell survival and tumorigenic potential, highlighting it as a potential target for antitumor therapy.	(296)
Nrf2/HO-1/GPX4	PPI inhibits hepatocellular carcinoma (HCC) growth by inducing ferroptosis through the modulation of the Nrf2/HO-1/GPX4 antioxidant axis and causing mitochondrial dysfunction, with effects comparable to those of sorafenib.	(232)
circFAM134B	circFAM134B as a regulator of ER-phagy-mediated ferroptosis in hepatocellular carcinoma (HCC) by sponging PABPC4 to stabilize FAM134B mRNA, enhancing the effectiveness of lenvatinib treatment through increased ROS and Fe2+ levels.	(297)
METTL9	METTL9 promotes hepatocellular carcinoma progression by inhibiting ferroptosis through upregulation of SLC7A11, with its knockdown significantly reducing tumor viability, migration, and invasion, suggesting METTL9 as a potential therapeutic target for HCC.	(224)
CircPIAS1	CircPIAS1 promotes hepatocellular carcinoma progression by inhibiting ferroptosis through the miR-455-3p/NUPR1/FTH1 axis, with NUPR1 inhibition sensitizing HCC cells to lenvatinib.	(298)
VDAC2	Celastrol targets VDAC2 to induce mitochondria-dependent ferroptosis and apoptosis in hepatocellular carcinoma, with liposome-based delivery enhancing its efficacy and reducing side effects.	(299)
ERK	Sorafenib induces ferroptosis in hepatocellular carcinoma by promoting TRIM54-mediated ubiquitination and degradation of FSP1 via the ERK pathway, with FSP1 reducing sorafenib sensitivity and enhancing tumor development.	(300)
FGF21	FGF21 promotes ferroptosis in hepatocellular carcinoma by upregulating Major vault protein (MVP), which enhances NOX4-mediated ROS production through IRF1 and YAP1 interactions.	(301)
Circ_0016142	Circ_0016142 promotes hepatocellular carcinoma cell proliferation by inhibiting ferroptosis through the miR-188-3p/GPX4 axis.	(31)
FABP5	FABP5 drives obesity-induced hepatocellular carcinoma by promoting lipid peroxidation resistance and immunosuppression, while its inhibition induces ferroptosis and a pro-inflammatory tumor microenvironment, offering a potential therapeutic strategy.	(302)
MerTK	MerTK drives anti-PD-1/PD-L1 resistance in hepatocellular carcinoma by limiting ferroptosis and promoting an immunosuppressive microenvironment, with its inhibition by sitravatinib enhancing therapy efficacy.	(303)
HO-1	ST ethanol extract induces ferroptosis in hepatoma cells through HO-1 expression and GPX4 suppression, with enhanced efficacy when combined with lenvatinib, even in resistant cells.	(304)
URB1-AS1	URB1-AS1 lncRNA suppresses ferroptosis in sorafenib-resistant hepatocellular carcinoma by reducing free iron content, with its inhibition enhancing sorafenib sensitivity, offering a potential strategy to overcome resistance.	(305)
FSP1	Ginsenoside RK1 induces ferroptosis in hepatocellular carcinoma cells by depleting GSH and increasing MDA and iron levels, with its effects modulated by Ferroptosis suppressor protein 1 (FSP1).	(306)
DUSP4	DUSP4 suppresses ferroptosis in hepatocellular carcinoma by regulating ferritin mRNA localization through YTHDC1 phosphorylation, contributing to sorafenib resistance.	(307)
HMOX1	F30, a FGFR4-targeting compound, induces ferroptosis in hepatocellular carcinoma cells by dysregulating iron levels and redox balance, with its effects dependent on HMOX1.	(308)
HRAS	HRAS promotes hepatocellular carcinoma progression by upregulating HSPB1, reducing ferroptosis, and enhancing cell proliferation and invasion.	(309)
–	Arvanil induces ferroptosis in hepatocellular carcinoma cells by increasing mitochondrial calcium flow and enhancing cisplatin chemosensitivity, offering potential as a therapeutic candidate for HCC.	(310)
SOX8	SOX8 overexpression triggers ferroptosis in hepatocellular carcinoma by disrupting glycolipid metabolism, redox balance, and iron homeostasis, suggesting a novel therapeutic strategy for HCC.	(311)
–	Celastrol inhibits hepatocellular carcinoma cell proliferation by inducing ferroptosis, with RRM2 playing a key role in modulating its effects on tumor growth and ferroptosis.	(312)
HAND2-AS1	HAND2-AS1 reverses lenvatinib resistance in hepatocellular carcinoma by promoting ferroptosis through the TLR4/NOX2/DUOX2 pathway via miR-219a-1-3p, with low HAND2-AS1 levels linked to early recurrence.	(313)
IL1β	Ferroptosis in hepatocellular carcinoma promotes tumor growth and metastasis through a macrophage/IL1β/neutrophil axis, which can induce sorafenib resistance, with targeting this inflammatory pathway enhancing sorafenib efficacy.	(314)
–	An oral nanoparticle platform combining sorafenib and salinomycin enhances ferroptosis and immune response in hepatocellular carcinoma, improving therapeutic efficacy by targeting HCC cells through MCT-1-mediated delivery.	(315)
JNK1-FOXQ1-ETHE1	The JNK1-FOXQ1-ETHE1 axis suppresses sorafenib-induced ferroptosis in hepatocellular carcinoma by reducing lipid peroxidation and iron levels, contributing to sorafenib resistance.	(316)
TfR1	CHIP acts as an oncogene in hepatocellular carcinoma by promoting cell proliferation and inhibiting ferroptosis through the degradation of transferrin receptor 1 (TfR1).	(317)
–	The pH-responsive nanocarrier HDP co-delivers sorafenib and siNRF2 to overcome NRF2-mediated ferroptosis resistance, significantly enhancing anti-tumor effects in hepatocellular carcinoma.	(318)
ATF3/HMOX1/GPX4	SEH1L promotes hepatocellular carcinoma progression by inhibiting ferroptosis, while its silencing induces ferroptosis via the ATF3/HMOX1/GPX4 axis, suppressing tumor growth.	(319)
–	Combining atovaquone with TCR-T cell therapy enhances ferroptosis and anti-tumor immune responses in hepatocellular carcinoma, improving treatment efficacy.	(320)
TBRG4	TBRG4 promotes hepatocellular carcinoma progression by inhibiting ferroptosis through the DDX56/p-AKT/GSK3β pathway and binding to Beclin1, while its knockdown reduces tumor cell proliferation, migration, and invasion.	(321)
PVT1	PLAG1, regulated by the lncRNA PVT1, confers resistance to sorafenib-induced ferroptosis in hepatocellular carcinoma by upregulating GPX4 and maintaining redox homeostasis.	(322)
PD-L1	IFN-γ exposure in the tumor microenvironment enhances liver cancer stem cell stemness and sorafenib resistance by promoting mitochondrial PD-L1-mediated glycolytic reprogramming and ferroptosis inhibition via the Drp1-GPX4 pathway.	(323)
STAT1	SM suppresses hepatocellular carcinoma growth by reducing STAT1-mediated MTCH1 expression, thereby inducing apoptosis and ferroptosis.	(324)
SNRPB	SNRPB promotes hepatocellular carcinoma progression by regulating immune checkpoints, cell cycle, oxidative stress, and ferroptosis, with its knockdown enhancing sorafenib’s therapeutic effect.	(325)
MARCH8	O-GlcNAcylation of TFRC regulates ferroptosis in hepatocellular carcinoma by enhancing TFRC stability and promoting iron accumulation through decreased MARCH8-mediated ubiquitination.	(326)
SNHG1	The SNHG1-miR-199a-FANCD2/G6PD axis inhibits ferroptosis in hepatocellular carcinoma, serving as a potential marker for prognosis and therapy.	(327)
ABHD12	ABHD12 promotes tumor growth and sorafenib resistance in liver cancer, with co-delivery of sorafenib and ABHD12 inhibitor enhancing therapeutic efficacy.	(328)
EZH2	EZH2 suppresses ferroptosis in HCC by downregulating TFR2, and combining EZH2 inhibitor tazemetostat with sorafenib enhances sorafenib sensitivity and anti-cancer effects.	(329)

## 12 The interplay of autophagy and ferroptosis in the tumor immune microenvironment of hepatocellular carcinoma

Autophagy and ferroptosis play complex and interrelated roles in the tumor immune microenvironment of liver cancer. Autophagy, a cellular degradation and recycling mechanism, can both provide necessary nutrients and energy to tumor cells under nutrient-deficient and stressful conditions, promoting their survival, and induce tumor cell death by degrading oncogenic proteins or damaged organelles, thereby inhibiting tumor growth (330, 331). Additionally, autophagy regulates immune cell functions, enhancing or suppressing immune responses. For example, it can boost the antigen-presenting ability of dendritic cells or promote immune evasion of tumor cells by modulating immune checkpoint expression. Autophagy also influences tumor progression and the immune microenvironment by regulating inflammatory responses and cytokine secretion (332). Ferroptosis, a form of cell death induced by iron-dependent lipid peroxidation, involves triggering iron-dependent lipid peroxidation within cells, leading to membrane damage and cell death, serving as an anti-tumor strategy to inhibit liver cancer progression. The occurrence of ferroptosis can release tumor antigens and inflammatory mediators, activating anti-tumor immune responses. It promotes dendritic cell maturation and antigen presentation, activating T cells’ anti-tumor activity, and alters the tumor microenvironment to attract immune cell infiltration, enhancing local immune responses. Additionally, ferroptosis can disrupt tumor cells’ immune evasion mechanisms by reducing the expression of immune checkpoint proteins on tumor cell surfaces, increasing their susceptibility to immune attacks (333, 334). In summary, autophagy and ferroptosis complement each other in the tumor immune microenvironment of liver cancer. Autophagy exerts dual roles under different conditions, promoting tumor cell survival and inhibiting tumor growth, while ferroptosis primarily inhibits tumor progression by inducing tumor cell death and activating immune responses. In future liver cancer treatments, modulating the mechanisms of autophagy and ferroptosis may become a new therapeutic strategy, capable of directly killing tumor cells and enhancing the body’s anti-tumor immune responses.

CISD2 exhibited higher expression in HCC cells than in normal cells and correlated with poorer patient prognosis (335). Reducing CISD2 levels decreased the viability of drug-resistant HCC cells and enhanced their sensitivity to sorafenib-induced ferroptosis. Increases in ROS, MDA, and iron ions were observed following CISD2 knockdown, while GSH levels remained stable. This knockdown also caused uncontrolled autophagy in the resistant HCC cells, which was mitigated when autophagy was inhibited, reducing the ferroptosis. The autophagy associated with CISD2 reduction was linked to Beclin1, and inhibiting both CISD2 and Beclin1 diminished the ferroptotic effects. ZFP36 is modulated by the ubiquitin ligase FBXW7/CDC4, which decreases ZFP36 levels, thereby facilitating ferroptosis (336). ZFP36 disrupts this process by destabilizing ATG16L1 mRNA, an essential autophagy-related molecule, effectively blocking ferroptosis. Enhancing ATG16L1 expression can counteract the effects of ZFP36, supporting autophagy and subsequent ferroptosis. Furthermore, genetic alterations that prevent ZFP36 from binding to ATG16L1 mRNA remove its ability to stabilize the mRNA and resist ferroptosis. Clinically, treatments like erastin and sorafenib, which induce ferroptosis in HSCs, have been effective in reducing liver fibrosis in mouse models and in patients with hepatocellular carcinoma undergoing sorafenib treatment. This highlights the therapeutic potential of targeting ZFP36-mediated pathways in liver disease management.

## 13 Conclusion

This review examines the development of both ferroptosis- and autophagy-related concepts in HCC and their application in treatment. Ferroptosis is crucial in cancers, particularly HCC, with sorafenib causing ferroptosis that can be inhibited by Rb, NRF2, and MT-IG. Haloperidol promotes sorafenib-induced ferroptosis, offering a combination therapy strategy. CISD1 is a negative regulator in HCC, providing insight into iron metabolism. Lipid metabolism is also critical for ferroptosis in HCC, with LDL–DHA causing ferroptosis and ACSL4 being a monitor and contributor. Progress has been made in understanding ferroptosis in HCC, but detailed signal transduction pathways and key transcriptional regulators remain unknown. Moreover, autophagy plays a double role in HCC, inhibiting tumors and promoting the survival of HCC cells in the tumor microenvironment. Understanding the specific molecular mechanisms of autophagy in different stages of HCC can help develop therapeutic targets and overcome resistance to current therapies. Novel treatment strategies to reduce HCC metastasis and resistance may include targeted medicines that affect autophagy.

The paper adeptly examines the dual roles of autophagy and ferroptosis in the context of HCC, offering a comprehensive overview of the molecular mechanisms and pathways that regulate these processes. The interplay between non-coding RNAs and these cell death mechanisms highlights a complex regulatory network that can be harnessed to develop targeted therapies. The use of traditional Chinese medicine and novel drug targets to modulate these pathways showcases an integrative approach that blends conventional and contemporary cancer treatments, potentially opening new avenues for overcoming drug resistance in HCC. The synthesis of detailed biochemical pathways and clinical implications presents a thorough narrative that not only deepens the understanding of HCC’s pathological basis but also bridges the gap between bench research and bedside application. The exploration of signaling pathways such as PI3K/AKT/mTOR and the roles of miRNAs and lncRNAs in influencing autophagy and ferroptosis establishes a framework for targeted genetic and pharmacological interventions.

Future developments in the field could see the rise of genetic editing tools like CRISPR/Cas9 being employed to directly modify the genes involved in autophagy and ferroptosis pathways. By correcting or silencing specific oncogenes or tumor suppressor genes, researchers could develop more precise therapies that could prevent the progression of HCC or enhance the efficacy of existing treatments. The integration of artificial intelligence and machine learning in the analysis of big data from genomics and proteomics could lead to the identification of novel biomarkers and therapeutic targets for HCC. AI algorithms could help in designing personalized medicine approaches by predicting patient responses to certain therapies based on their genetic makeup and disease phenotype. The application of nanotechnology for drug delivery presents a promising avenue to improve the specificity and effectiveness of therapies targeting autophagy and ferroptosis. Nanoparticles could be engineered to deliver drugs directly to tumor cells, reducing side effects and improving drug stability and absorption. Exploring the interaction between cell death mechanisms and the immune system could lead to the development of novel immunotherapies. For instance, drugs that induce ferroptosis could be combined with immune checkpoint inhibitors to enhance the immunogenicity of cancer cells, making them more susceptible to immune-mediated destruction. More comprehensive and multicentric clinical trials will be essential to validate the safety and efficacy of new treatments that arise from this research. These studies will need to include diverse patient populations to ensure that the findings are widely applicable and effective across different genetic backgrounds and environmental factors. As new therapies are developed, particularly those involving genetic manipulation or novel drug compounds, regulatory frameworks will need to be adapted to ensure that these innovations are safely integrated into clinical practice. Ethical considerations, especially in genetic editing, will also require rigorous discussion and consensus. In the recent years, nanoparticles have also gained much attention in the treatment of human cancers (337). Therefore, the regulation of autophagy and ferroptosis by nanostructures in the treatment of HCC should be followed with more details.

In HCC, autophagy is intricately regulated by a series of interconnected pathways that include the PI3K/AKT/mTOR and AMPK/mTOR pathways, where activation of PI3K and AKT leads to mTOR inhibition, promoting autophagy under stress conditions. The autophagic process is initiated by the ULK1 complex and the Beclin-1 complex, which includes Class III PI3K and Vps34, essential for autophagosome formation. This process is further facilitated by autophagy-related (Atg) proteins that aid in the elongation and maturation of autophagosomes, crucial for cell survival, proliferation, and chemotherapy resistance. Conversely, ferroptosis in HCC is a regulated cell death mechanism driven primarily by iron metabolism and lipid peroxidation, which involves the accumulation of lipid peroxides that are catalyzed by cellular iron. The system Xc− plays a pivotal role by importing cystine for glutathione synthesis, which is used by GPX4 to reduce lipid peroxides, thus preventing ferroptosis. Additionally, the p53 signaling pathway can enhance ferroptosis by suppressing system Xc−, and the NRF2 pathway contributes to antioxidant defenses that inhibit ferroptosis. Interestingly, there is significant cross-talk between autophagy and ferroptosis; for instance, autophagy can promote ferroptosis by degrading ferritin, releasing free iron, and increasing oxidative stress within the cell, highlighting a complex interplay that influences HCC progression and treatment response.

HCC is characterized by a remarkable genetic diversity, which is influenced by a myriad of factors including genetic mutations, epigenetic modifications, and varying environmental exposures such as viral infections (particularly hepatitis B and C viruses), alcohol consumption, and aflatoxin exposure. These factors contribute to the diverse molecular landscapes of HCC, affecting the activation and inhibition of pathways like autophagy and ferroptosis differently in different patient populations. For example, genetic mutations in key regulators of autophagy such as ATG5 or BECN1 and alterations in the expression of components of the ferroptosis pathway like GPX4 and SLC7A11 can affect the susceptibility of HCC cells to therapies that target these pathways. Moreover, epigenetic modifications, including DNA methylation and histone modifications, can lead to the silencing or activation of genes involved in autophagy and ferroptosis, further complicating the therapeutic landscape. Environmental factors also interact with genetic and epigenetic mechanisms, influencing the metabolic and oxidative stress states within the liver, thereby modulating the effectiveness of treatments aimed at inducing specific types of cell death. Addressing this heterogeneity in future studies and reviews could enhance the understanding of why certain therapies may succeed or fail in different subgroups of HCC patients. It would be advantageous to integrate genomic, epigenomic, and environmental data in clinical trials to develop more personalized approaches in the treatment of HCC. This could potentially lead to the identification of biomarkers that predict response to therapies targeting autophagy and ferroptosis, ultimately improving patient outcomes by tailoring treatments according to individual genetic and environmental backgrounds.
